# Supplementary material for: Patient and caregiver characteristics associated with differential use of primary care for children and young people in the UK: a scoping review
Source: BMJ Open. 2024 May 16;14(5):e078505. doi: 10.1136/bmjopen-2023-078505 (PMC11103219; doi:10.1136/bmjopen-2023-078505)
Supplement: Supplementary data [file bmjopen-2023-078505supp001.pdf]

Patient and caregiver characteristics associated with differential use of primary care for children and young people in the UK: a scoping review

Appendices

Appendix A: Search terms and results

Ovid

- Embase 1996 to 2022 Week 24
- Ovid MEDLINE(R) 1996 to June Week 2 2022

Search conducted 23rd June 2022

Search		Terms	Hits
**1**	Service access	exp "Health Services Accessibility"/ or "access to health services".tw. or "access to care".tw. or exp "Health Equity"/ or "health equity".tw. or exp "Health Literacy"/ or "health literacy".tw. or "healthcare use".tw. or "healthcare utilisation".tw. or "healthcare utilization".tw. or inequality.tw. or inequalities.tw. or equality.tw. or disparity.tw. or disparities.tw. or unequal.tw. or gap*.tw. or gradients.tw. or disadvantage.tw.	1,076,198
**2**	Primary care	exp "Primary Health Care"/ or "primary health care".tw. or "primary care".tw. or exp "Primary Care Nursing"/ or exp "Physicians, Primary Care"/ or exp "General Practice"/ or "general practice*".tw. or "GP surger*".tw. or exp "General Practice, Dental"/ or dentist*.tw. or exp "General Practitioners"/ or "general practitioner*".tw. or exp "Community Pharmacy Services"/ or "community pharmac*".tw.	766,436
**3**	CYP	exp Child/ or child*.tw. or exp Adolescent/ or adolescen*.tw. or exp Infant/ or infant*.tw. or "young people".tw. or youth.tw. or juvenile*.tw. or teenager*.tw. or exp "Parent-Child Relations"/ or "young adult*".tw. or paediatri*.tw.	6,208,476
**4**	UK	("United Kingdom" or England or Wales or Scotland or "Northern Ireland" or "N. Ireland").af.	13,100,534
**5**	Quantitiative studies	(quantit* or consultation* or episode* or visit* or longitudin* or patient-level).af.	4,071,128
**6**		1 and 2 and 3 and 4 and 5	1,932
**7**	Limit to human studies	limit 6 to humans	1,915
**8**	Limit to years 2010 - 2022	limit 7 to yr="2010 - 2022"	1,427
**9**	Deduplication<br><br>Preferences:	remove duplicates from 8<br><br>Deduplication (Ovid)<br>Has full text; Embase > Ovid MEDLINE(R)	1,097

Herbert K, *et al. BMJ Open* 2024; 14:e078505. doi: 10.1136/bmjopen-2023-078505

Scopus

Search conducted 23rd June 2022

	Search	Terms	Hits
**1**	Service access	INDEXTERMS("Health Services Accessibility") OR TITLE-ABS("access to health services") OR TITLE-ABS("access to care") OR INDEXTERMS("Health Equity") OR TITLE-ABS("health equity") OR INDEXTERMS("Health Literacy") OR TITLE-ABS("health literacy") OR TITLE-ABS("healthcare use") OR TITLE-ABS("healthcare utilisation") OR TITLE-ABS("healthcare utilization") OR TITLE-ABS(inequality) OR TITLE-ABS(inequalities) OR TITLE-ABS(equality) OR TITLE-ABS(disparity) OR TITLE-ABS(disparities) OR TITLE-ABS(unequal) OR TITLE-ABS(gap*) OR TITLE-ABS(gradients) OR TITLE-ABS(disadvantage)	2,671,314
**2**	Primary care	INDEXTERMS("Primary Health Care") OR TITLE-ABS("primary health care") OR TITLE-ABS("primary care") OR INDEXTERMS("Primary Care Nursing") OR INDEXTERMS("Physicians, Primary Care") OR INDEXTERMS("General Practice") OR TITLE-ABS("general practice*") OR TITLE-ABS("GP surger*") OR INDEXTERMS("General Practice, Dental") OR TITLE-ABS(dentist*) OR INDEXTERMS("General Practitioners") OR TITLE-ABS("general practitioner*") OR INDEXTERMS("Community Pharmacy Services") OR TITLE-ABS("community pharmac*")	481,759
**3**	CYP	INDEXTERMS(Child) OR TITLE-ABS(child*) OR INDEXTERMS(Adolescent) OR TITLE-ABS(adolescen*) OR INDEXTERMS(Infant) OR TITLE-ABS(infant*) OR TITLE-ABS("young people") OR TITLE-ABS(youth) OR TITLE-ABS(juvenile*) OR TITLE-ABS(teenager*) OR INDEXTERMS("Parent-Child Relations") OR TITLE-ABS("young adult*") OR TITLE-ABS(paediatri*)	5,664,633
**4**	UK	ALL("United Kingdom") OR ALL(England) OR ALL(Wales) OR ALL(Scotland) OR ALL("Northern Ireland") OR ALL("N. Ireland")	12,674,770
**5**	Quantitiative studies	ALL(quantit* OR consultation* OR episode* OR visit* OR longitudin* OR patient-level)	11,926,797
**6**	Combine 1-5	#1 AND #2 AND #3 AND #4 AND #5	2,018
**7**	Limit to articles or reviews	LIMIT-TO (DOCTYPE, "ar") OR LIMIT-TO (DOCTYPE, "re")	1,958
**8**	Limit to years 2010 - 2022	LIMIT-TO (PUBYEAR, 2022) OR LIMIT-TO (PUBYEAR, 2021) OR LIMIT-TO (PUBYEAR, 2020) OR LIMIT-TO (PUBYEAR, 2019) OR LIMIT-TO (PUBYEAR, 2018) OR LIMIT-TO (PUBYEAR, 2017) OR LIMIT-TO (PUBYEAR, 2016) OR LIMIT-TO (PUBYEAR, 2015) OR LIMIT-TO (PUBYEAR, 2014) OR LIMIT-TO (PUBYEAR, 2013) OR LIMIT-TO (PUBYEAR, 2012) OR LIMIT-TO (PUBYEAR, 2011) OR LIMIT-TO (PUBYEAR, 2010)	1,372
**9**	Limit to English language publications	LIMIT-TO (LANGUAGE,"English")	1,354

Herbert K, *et al. BMJ Open* 2024; 14:e078505. doi: 10.1136/bmjopen-2023-078505

Web of Science

Web of Science Core Collection: All editions

Search conducted 22nd June 2022

	Search	Terms	Hits
1	Service access	ALL=("Health Services Accessibility" OR "access to health services" OR "access to care" OR "Health Equity" OR "health equity" OR "Health Literacy" OR "health literacy" OR "healthcare use" OR "healthcare utilisation" OR "healthcare utilization" OR inequality OR inequalities OR equality OR disparity OR disparities OR unequal OR gap* OR gradients OR disadvantage)	2,284,588
2	Primary care	ALL=("Primary Health Care" OR "primary health care" OR "primary care" OR "Primary Care Nursing" OR "Physicians, Primary Care" OR "General Practice" OR "general practice*" OR "GP surger*" OR "General Practice, Dental" OR dentist* OR "General Practitioners" OR "general practitioner*" OR "Community Pharmacy Services" OR "community pharmac*")	444,274
3	CYP	ALL=(Child OR child* OR Adolescent OR adolescen* OR Infant OR infant* OR "young people" OR youth OR juvenile* OR teenager* OR "Parent-Child Relations" OR "young adult*" OR paediatri*)	4,378,563
4	UK	ALL=("United Kingdom" OR England OR Wales OR Scotland OR "Northern Ireland" OR "N. Ireland")	6,796,242
5	Quantitiative studies	ALL=(quantit* OR consultation* OR episode* OR visit* OR longitudin* OR patient-level)	3,328,555
6		#1 AND #2 AND #3 AND #4 AND #5	408
7	Limit to years 2010 - 2022	#6<br>Publication date: 2010-01-01 to 2022-01-01	315
8	Limit to articles or reviews	#7 AND DT=(Article OR Review)	313
9	Limit to English language publications	(#8) AND LA=(English)	312

Google

Grey literature search

Search conducted 5th July 2022

Search terms

(site:.org.uk OR site:.gov.uk) AND General Practice* OR General Practitioner* OR Primary Care OR GP* AND Inequality OR Inequalities

Herbert K, *et al. BMJ Open* 2024; 14:e078505. doi: 10.1136/bmjopen-2023-078505

Appendix B: Characteristics of included studies (Healthcare need indicator variables for the characteristics of CYP and their caregivers in bold).

**Author (year)** ***sample location***	**Design**	**Health care setting; primary focus of study**	**Sample population**	**Age of sample**	**CYP/caregiver characteristics examined**		**MMAT Score**
					**Sample subset(s)**	**Controlling variable(s)**	
Coughlan *et al.* (2022) *England*	Retrospective cohort study	*General practice, outpatients, emergency department, hospital admissions*: Differences in health care use by health care setting, level of deprivation and ethnic group.	1,484,455 (from 408 GP practices)	0 – 14 years	Ethnicity, deprivation (IMD)	-	****
Beaney *et al.* (2021) *England*	Retrospective cohort study	*General practice, outpatients, emergency department, hospital admissions*: Patterns of healthcare utilisation in the CYP population in Northwest London	378,309	0 – 15 years	**Age**, sex, ethnicity, deprivation (IMD)	-	***** §
de Lusignan *et al.* (2021) *England*	Population-based cohort study	*General practice, specialist referrals:* Use of services and treatment for eczema patients	53,780	0 – 18 years	**Age**, sex, ethnicity, deprivation (IMD), rural-urban classification	-	***** §
Hasan & West (2021) *England*	Prospective cohort study	*Primary care consultations (not specified):* Effects of obesity and ethnicity	7,815 (3,469 White British, 4,346 Pakistani)	8 years	Ethnicity	**Age**, sex, ethnicity, deprivation (not specified), **birthweight**, **gestational age**, maternal BMI, maternal age	*****
Hope *et al.* (2021) *England*	Retrospective cohort study	*General practice, outpatients, emergency department, hospital admissions*: Children exposed to maternal mental illness	489,255	0 – 17 years	**Age**, Ethnicity	Deprivation (IMD)	***** §
Manikam *et al.* (2020) *England*	Retrospective cohort study	*General practice, hospital admissions*: Respiratory tract infection-related healthcare utilisation in children with Down's syndrome	5,866 (992, Down's syndrome, 4,874 control)	0 – 18 years	**Age**, sex, **comorbidities**, **respiratory tract infection type**	-	***** §
Perry *et al.* (2020) *Wales*	Routine health data linked to birth cohort	*General practice (vaccinations)*: Association of deprivation with pertussis vaccination uptake	163,733	0 – 1 years	Deprivation (Welsh IMD)	-	****
Ruzangi *et al.* (2020) *England*	Population-based cohort study	*General practice, outpatients, emergency department, hospital admissions*: Analysis of individual-level patient data to derive population-based estimates of healthcare utilisation	1,484,455 (from 408 GP practices)	0 – 14 years	**Age**	-	***** §
Edbrooke-Childs & Patalay (2019) *England*	Routine youth mental health services data	*General practice, emergency department, mental health services, child health services, education services*: Referral routes to youth mental health services	14,588	0 – 25 years (0-5, 6-12, 13-25 years)	-	**Age**, sex, ethnicity, deprivation (IDACI), **problem type**, contextual factors (home life, school, community, engagement)	*****
Salomon-Ibarra *et al.* (2019) *England*	Routine health data linked to birth cohort	*NHS England dental services:* Dental attendance	155,308	0 – 1 year	-	Deprivation (IMD)	****

Herbert K, *et al. BMJ Open* 2024; 14:e078505. doi: 10.1136/bmjopen-2023-078505

Bishop *et al.* (2018) *England*	Prospective birth cohort study	*General practice, outpatients, emergency department, hospital admissions*: Children with congenital abnormalities on utilisation of primary and secondary care and referrals to specialists.	13,857	0 – 4 years	-	Deprivation (Means-tested benefit status), ethnicity, maternal age, maternal educational attainment, consanguinity, **multimorbidity**	*****
Dreyer *et al.* (2018) *England*	Retrospective, cross-sectional study	*General practice, outpatients, emergency department, hospital admissions*: Parental mental health and other family factors	25,252	0 – 15 years	-	**Age**, sex, deprivation (IMD), **CYP long-term conditions** Parent: age, healthcare utilisation, long-term conditions Household: Number of adults aged 18–55 years, number of CYP aged 0–15 years, presence of any patient aged ≥65 years, total number of patients in household	*****
West *et al.* (2018) *England*	Prospective cohort study	*General practice, outpatients, emergency department, hospital admissions*: Early childhood morbidity and small size at birth	8,850	0 – 3 years	Ethnicity	Sex, maternal parity, **gestational age**, maternal age, social economic information (maternal education, housing tenure, means-tested bene- fits), smoking	*****
Cecil *et al.* (2016) *England*	Cross-sectional, population-based study	*General practice, emergency department, hospital admissions*: Primary care access, emergency department visits, and unplanned short hospitalizations	9.5 million	0 – 15 years	-	Practice deprivation (IMD), urban/rural profile, percentage of the registered population aged ≥65 years	****
Edbrooke-Childs *et al.* (2016) *England*	Routinely collected child and adolescent mental health services data	*General practice, emergency department, social services, education services, child health services, youth justice, self-referral, other services:* Children with mental health problems	11,592 (26 CAMHS)	0 – 18 years	-	**Age**, sex, ethnicity	*****
Layte & Nolan (2015) *Ireland and Scotland*	Child cohort studies	*General practice:* Use of GP services	9,719	2 and 4 years	-	**Age**, sex, deprivation (family income) Healthcare need: **Birthweight**, **gestation**, **parental assessment of child's general health status**, **exposure to accidents** Non-need: Number of siblings, maternal highest level of education, maternal employment status, household composition, maternal ethnicity Health care entitlements	*****

Herbert K, *et al. BMJ Open* 2024; 14:e078505. doi: 10.1136/bmjopen-2023-078505

Alkahtani *et al.* (2014) *England*	Cross-sectional study	*General practice, over the counter medication:* Access to medicines by child refugees	216 (117 refugee, 99 control)	4 – 5 years	-	-	***
Vostanis *et al.* (2013) *England* †	Cross-sectional study	*General practice, CAMHS and paediatric hospital services*: Difference in level of need and service utilisation by young ethnic minority groups.	2,900	13 – 15 years	-	**Age**, ethnicity, free school meals, abnormal score (SDQ or MFQ-short), who the child lives with	*****
Marcenes *et al.* (2013) *England*	Cluster sampled, cross-sectional study	*Dental health (British Association for the Study of Community Dentistry):* Oral health	1,285	3 – 4 years	Deprivation (IMD)	Sex, ethnicity, London borough	****
Potter *et al.* (2012) *UK*	Longitudinal cohort study	General practice, paediatrics, mental health services, social services, youth justice: Mental disorder in children of parents with depression	333	9 – 17 years	**Age**, sex, parental age, family status, parental problem drinking, **child comorbidity (≥2 disorders)**, **child suicidality**/ **self-harm**, lack of parental concern, parental DSM-IV current depressive episode, frequent parental depressive episodes (≥4)	-	**** §
Telford & O'Neill (2012) *Northern Ireland*	Longitudinal cohort study	*General Dental Service (Northern Ireland):* Dental health investment	13,564	11 – 12 years	**Age**	Sex, deprivation (NS-SEC), highest educational attainment, parental marital status, number of siblings	***** §
Telford *et al.* (2012) *Northern Ireland*	Longitudinal cohort study	*General Dental Service (Northern Ireland):* Provision of NHS general dental practitioner care	12,846	11 – 12 years	-	Highest educational attainment, parental marital status, number of siblings, orthodontic treatment	****

† Identified from Markkula *et al.*, 2018.
‡ MMAT score: ***** high quality; **** high quality; *** average quality; ** low quality; * low quality; No stars, poor quality.
§ Indicator of healthcare need used to define study sample only.
Abbreviations: CAMHS, child and adolescent mental health services; CYP, children and young people; GP, general practice; IDACI, Income Deprivation Affecting Children Index; IMD, Index of Multiple Deprivation; MFQ-short, Mood and Feelings Questionnaire-short; MMAT, Mixed Methods Appraisal Tool; NHS, National Health Service; NS-SEC, National statistics socio-economic classification of occupation; SDQ, Strengths and Difficulties Questionnaire.

Herbert K, *et al. BMJ Open* 2024; 14:e078505. doi: 10.1136/bmjopen-2023-078505

Appendix C: Mixed Methods Appraisal Tool (MMAT) quality assessment of eligible studies

Criteria scoring: 0 = Not met; 1 = Partially met; 2 = Fully met.

Total score (Max 14): * ≤5; ** 6-9; *** 10-11; **** 12-13; ***** 14.

		Publication										
Category of study designs	Methodological quality criteria	Coughlan *et al.*, 2022	Beaney *et al.*, 2021	de Lusignan *et al.*, 2021	Hasan & West, 2021	Hope *et al.*, 2021	Manikam *et al.*, 2020	Perry *et al.*, 2020	Ruzangi *et al.*, 2020	Edbrooke-Childs & Patalay, 2019	Salomon-Ibarra *et al.*, 2019	Bishop *et al.*, 2018
Screening questions (for all types)	S1. Are there clear research questions?	2	2	2	2	2	2	2	2	2	2	2
	S2. Do the collected data allow to address the research questions?	2	2	2	2	2	2	2	2	2	2	2
Further appraisal may not be feasible or appropriate when the answer is 'No' or 'Can't tell' to one or both screening questions.												
Quantitative non-randomized controlled trials	3.1. Are the participants representative of the target population?					2						2
	3.2. Are measurements appropriate regarding both the outcome and intervention (or exposure)?					2						2
	3.3. Are there complete outcome data?					2						2
	3.4. Are the confounders accounted for in the design and analysis?					2						2
	3.5. During the study period, is the intervention administered (or exposure occurred) as intended?					2						2
Quantitative descriptive	4.1. Is the sampling strategy relevant to address the research question?	2	2	2	2		2	2	2	2	2	
	4.2. Is the sample representative of the target population?	2	2	2	2		2	2	2	2	2	
	4.3. Are the measurements appropriate?	2	2	2	2		2	2	2	2	2	
	4.4. Is the risk of nonresponse bias low?	2	2	2	2		2	2	2	2	2	
	4.5. Is the statistical analysis appropriate to answer the research question?	2	2	2	2		2	2	2	2	2	
Total score		14	14	14	14	14	14	14	14	14	14	14

Herbert K, *et al. BMJ Open* 2024; 14:e078505. doi: 10.1136/bmjopen-2023-078505

		Publication										
Category of study designs	Methodological quality criteria	Dreyer *et al.*, 2018	Vostanis et al., 2013 †	West *et al.*, 2018	Cecil *et al.*, 2016	Edbrooke-Childs *et al.*, 2016	Layte & Nolan, 2015	Alkahtani *et al.*, 2014	Marcenes *et al.*, 2013	Potter *et al.*, 2012	Telford & O'Neill, 2012	(C. Telford *et al.*, 2012)
Screening questions (for all types)	S1. Are there clear research questions?	2	2	2	2	2	2	2	2	2	2	2
	S2. Do the collected data allow to address the research questions?	2	2	2	2	2	2	2	2	2	2	2
Further appraisal may not be feasible or appropriate when the answer is 'No' or 'Can't tell' to one or both screening questions.												
Quantitative non-randomized controlled trials	3.1. Are the participants representative of the target population?				2		2	1				
	3.2. Are measurements appropriate regarding both the outcome and intervention (or exposure)?				2		2	2				
	3.3. Are there complete outcome data?				2		2	2				
	3.4. Are the confounders accounted for in the design and analysis?				2		2	0				
	3.5. During the study period, is the intervention administered (or exposure occurred) as intended?				2		2	2				
Quantitative descriptive	4.1. Is the sampling strategy relevant to address the research question?	2	2	2		2			2	2	2	2
	4.2. Is the sample representative of the target population?	2	2	2		2			2	1	2	2
	4.3. Are the measurements appropriate?	2	2	2		2			2	2	2	2
	4.4. Is the risk of nonresponse bias low?	2	2	2		2			2	2	2	2
	4.5. Is the statistical analysis appropriate to answer the research question?	2	2	2		2			2	2	2	2
Total score		14	14	14	14	14	14	11	14	13	14	14

† Identified from Markkula *et al.*, 2018.

References

Alkahtani, S., Cherrill, J., Millward, C., Grayson, K., Hilliam, R., Sammons, H., & Choonara, I. (2014). Access to medicines by child refugees in the east midlands region of england: A cross-sectional study. *BMJ Open*, *4*(12). https://doi.org/10.1136/bmjopen-2014-006421

Beaney, T., Clarke, J., Woodcock, T., McCarthy, R., Saravanakumar, K., Barahona, M., Blair, M., & Hargreaves, D. S. (2021). Patterns of healthcare utilisation in children and young people: A retrospective cohort study using routinely collected healthcare data in Northwest London. *BMJ Open*, *11*(12). https://doi.org/10.1136/bmjopen-2021-050847

Bishop, C., Small, N., & Parslow, R. (2018). Healthcare use for children with complex needs: Using routine health data linked to a multiethnic, ongoing birth cohort. *BMJ Open*, *8*(3), e018419. https://doi.org/http://dx.doi.org/10.1136/bmjopen-2017-018419

Cecil, E., Bottle, A., Cowling, T. E., Majeed, A., Wolfe, I., & Saxena, S. (2016). Primary care access, emergency department visits, and unplanned short hospitalizations in the UK. *Pediatrics*, *137*(2). https://doi.org/10.1542/peds.2015-1492

Coughlan, C., Ruzangi, J., Neale, F., Nezafat Maldonado, B., Blair, M., Bottle, A., Saxena, S., & Hargreaves, D. (2022). Social and ethnic group differences in healthcare use by children aged 0-14 years: A population-based cohort study in England from 2007 to 2017. *Archives of Disease in Childhood*, *107*(1), 32–39. https://doi.org/https://dx.doi.org/10.1136/archdischild-2020-321045

de Lusignan, S., Alexander, H., Broderick, C., Dennis, J., McGovern, A., Feeney, C., & Flohr, C. (2021). Patterns and trends in eczema management in UK primary care (2009-2018): A population-based cohort study. *Clinical and Experimental Allergy*, *51*(3), 483–494. https://doi.org/http://dx.doi.org/10.1111/cea.13783

Dreyer, K., Williamson, R., Hargreaves, D., & Rosen, R. (2018). Associations between parental mental health and other family factors and healthcare utilisation among children and young people: A retrospective, cross-sectional study of linked healthcare data. *BMJ Paediatrics Open*, *2*(1), e000266. https://doi.org/https://dx.doi.org/10.1136/bmjpo-2018-000266

Edbrooke-Childs, J., Newman, R., Fleming, I., Deighton, J., & Wolpert, M. (2016). The association between ethnicity and care pathway for children with emotional problems in routinely collected child and adolescent mental health services data. *European Child and Adolescent Psychiatry*, *25*(5), 539–546. https://doi.org/10.1007/s00787-015-0767-4

Edbrooke-Childs, J., & Patalay, P. (2019). Ethnic Differences in Referral Routes to Youth Mental Health Services. *Journal of the American Academy of Child and Adolescent Psychiatry*, *58*(3), 368-375.e1. https://doi.org/https://dx.doi.org/10.1016/j.jaac.2018.07.906

Hasan, T., & West, J. (2021). Body mass index and use and costs of primary care services among white british and pakistani children: Findings from the born in bradford cohort study. *Archives of Disease in Childhood*, *106*(SUPPL 1), A393–A394. https://doi.org/http://dx.doi.org/10.1136/archdischild-2021-rcpch.683

Hope, H., Osam, C., Kontopantelis, E., Hughes, S., Munford, L., Ashcroft, D., & Pierce, M. (2021). The healthcare resource impact of maternal mental illness on children and adolescents: UK retrospective cohort study. *British Journal of Psychiatry*, *219*(3), 515–522. https://doi.org/https://dx.doi.org/10.1192/bjp.2021.65

Layte, R., & Nolan, A. (2015). Income-related inequity in the use of GP services by children: a comparison of Ireland and Scotland. *The European Journal of Health Economics : HEPAC : Health Economics in Prevention and Care*, *16*(5), 489–506.

https://doi.org/https://dx.doi.org/10.1007/s10198-014-0587-3

Manikam, L., Schilder, A., Lakhanpaul, M., Littlejohns, P., & Alexander, E. (2020). Respiratory tract infection-related healthcare utilisation in children with Down's syndrome. *Infection*, *48*(3), 403–410. https://doi.org/http://dx.doi.org/10.1007/s15010-020-01408-5

Marcenes, W., Muirhead, V. E., Murray, S., Redshaw, P., Bennett, U., & Wright, D. (2013). Ethnic disparities in the oral health of three- to four-year-old children in East London. *British Dental Journal*, *215*(2). https://doi.org/10.1038/sj.bdj.2013.687

Markkula, N., Cabieses, B., Lehti, V., Uphoff, E., Astorga, S., & Stutzin, F. (2018). Use of health services among international migrant children - a systematic review. *Globalization and Health*, *14*(1). https://doi.org/10.1186/s12992-018-0370-9

Perry, M., McGowan, A., Roberts, R., & Cottrell, S. (2020). Timeliness and equity of infant pertussis vaccination in wales: Analysis of the three dose primary course. *Vaccine*, *38*(6), 1402–1407. https://doi.org/https://dx.doi.org/10.1016/j.vaccine.2019.12.001

Potter, R., Mars, B., Eyre, O., Legge, S., Ford, T., Sellers, R., Craddock, N., Rice, F., Collishaw, S., Thapar, A., & Thapar, A. K. (2012). Missed opportunities: Mental disorder in children of parents with depression. *British Journal of General Practice*, *62*(600), e487–e493. https://doi.org/10.3399/bjgp12X652355

Ruzangi, J., Blair, M., Cecil, E., Greenfield, G., Bottle, A., & Hargreaves, D. (2020). Trends in healthcare use in children aged less than 15 years: A population-based cohort study in England from 2007 to 2017. *BMJ Open*, *10*(5), e033761. https://doi.org/http://dx.doi.org/10.1136/bmjopen-2019-033761

Salomon-Ibarra, C., Ravaghi, V., Hill, K., Jones, C., Landes, D., & Morris, A. (2019). Low rates of dental attendance by the age of one and inequality between local government administrative areas in England. *Community Dental Health*, *36*(1), 22–26. https://doi.org/10.1922/CDH_4390Salomon-Ibarra05

Telford, C. J., & O'Neill, C. (2012). Summary of: Changes in dental health investment across the adolescent years. *British Dental Journal*, *212*(9), 438–439. https://doi.org/10.1038/sj.bdj.2012.372

Telford, C., Murray, L., Donaldson, M., & O'Neill, C. (2012). An analysis examining socio-economic variations in the provision of NHS general dental practitioner care under a fee for service contract among adolescents: Northern Ireland Longitudinal Study. *Community Dentistry and Oral Epidemiology*, *40*(1), 70–79. https://doi.org/http://dx.doi.org/10.1111/j.1600-0528.2011.00649.x

Vostanis, P., Svirydzenka, N., Dugard, P., Singh, S., & Dogra, N. (2013). Mental health service use by adolescents of Indian and White origin. *Archives of Disease in Childhood*, *98*(10), 764 LP – 767. https://doi.org/10.1136/archdischild-2013-303772

West, J., Kelly, B., Collings, P., Santorelli, G., & Mason, D. (2018). Is small size at birth associated with early childhood morbidity in white British and Pakistani origin UK children aged 0-3? Findings from the born in Bradford cohort study. *BMC Pediatrics*, *18*(1), 22. https://doi.org/http://dx.doi.org/10.1186/s12887-018-0987-0
