# Supplementary material for: Patient and caregiver characteristics associated with differential use of primary care for children and young people in the UK: a scoping review
Source: BMJ Open. 2024 May 16;14(5):e078505. doi: 10.1136/bmjopen-2023-078505 (PMC11103219; doi:10.1136/bmjopen-2023-078505)
Supplement: Author's manuscript [file bmjopen-2023-078505.draft_revisions.pdf]

BMJ Open is committed to open peer review. As part of this commitment we make the peer review history of every article we publish publicly available.

When an article is published we post the peer reviewers' comments and the authors' responses online. We also post the versions of the paper that were used during peer review. These are the versions that the peer review comments apply to.

The versions of the paper that follow are the versions that were submitted during the peer review process. They are not the versions of record or the final published versions. They should not be cited or distributed as the published version of this manuscript.

BMJ Open is an open access journal and the full, final, typeset and author-corrected version of record of the manuscript is available on our site with no access controls, subscription charges or pay-per-view fees (http://bmjopen.bmj.com).

If you have any questions on BMJ Open's open peer review process please email info.bmjopen@bmj.com

Patient and caregiver characteristics associated with differential use of primary care for children and young people in the UK: a systematic scoping review

Journal:	*BMJ Open*
Manuscript ID	bmjopen-2023-078505
Article Type:	Original research
Date Submitted by the Author:	03-Aug-2023
Complete List of Authors:	Herbert, Kevin; Cambridge University, Public Health and Primary Care; Cambridge Research Methods Hub,<br>Herlitz, Lauren; UCL Great Ormond Street Institute of Child Health, Population<br>Woodman, Jenny; UCL, Social Research Institute<br>Powell, Claire; University College London, Institute of Child Health<br>Morris, Stephen; University of Cambridge, Primary Care Unit
Keywords:	Primary Care < Primary Health Care, Health Equity, Adolescent, Health Services Accessibility

SCHOLARONE™
Manuscripts

I, the Submitting Author has the right to grant and does grant on behalf of all authors of the Work (as defined in the below author licence), an exclusive licence and/or a non-exclusive licence for contributions from authors who are: i) UK Crown employees; ii) where BMJ has agreed a CC-BY licence shall apply, and/or iii) in accordance with the terms applicable for US Federal Government officers or employees acting as part of their official duties; on a worldwide, perpetual, irrevocable, royalty-free basis to BMJ Publishing Group Ltd ("BMJ") its licensees and where the relevant Journal is co-owned by BMJ to the co-owners of the Journal, to publish the Work in this journal and any other BMJ products and to exploit all rights, as set out in our licence.

The Submitting Author accepts and understands that any supply made under these terms is made by BMJ to the Submitting Author unless you are acting as an employee on behalf of your employer or a postgraduate student of an affiliated institution which is paying any applicable article publishing charge ("APC") for Open Access articles. Where the Submitting Author wishes to make the Work available on an Open Access basis (and intends to pay the relevant APC), the terms of reuse of such Open Access shall be governed by a Creative Commons licence – details of these licences and which Creative Commons licence will apply to this Work are set out in our licence referred to above.

Other than as permitted in any relevant BMJ Author's Self Archiving Policies, I confirm this Work has not been accepted for publication elsewhere, is not being considered for publication elsewhere and does not duplicate material already published. I confirm all authors consent to publication of this Work and authorise the granting of this licence.

For peer review only

Patient and caregiver characteristics associated with differential use of primary care for children and young people in the UK: a systematic scoping review

For peer review only

For peer review only - http://bmjopen.bmj.com/site/about/guidelines.xhtml

Patient and caregiver characteristics associated with differential use of primary care for children and young people in the UK: a systematic scoping review

Kevin Herbert, ORCID 0009-0008-4354-7811* Primary Care Unit, Department of Public Health & Primary Care, University of Cambridge, Cambridge, UK.

Lauren Herlitz, ORCID 0000-0003-2497-9041. Population, Policy and Practice, UCL Great Ormond Street Institute of Child Health, London, United Kingdom.

Jenny Woodman, ORCID 0000-0002-9403-4177. Thomas Coram Research Unit, UCL Social Research Institute, 27 Woburn Square, London, UK

Claire Powell, ORCID 0000-0002-6581-0165. Population, Policy and Practice, UCL Great Ormond Street Institute of Child Health, London, United Kingdom.

Stephen Morris, ORCID 0000-0002-5828-3563. Primary Care Unit, Department of Public Health & Primary Care, University of Cambridge, Cambridge, UK.

*Corresponding author

For peer review only - http://bmjopen.bmj.com/site/about/guidelines.xhtml

Abstract (max 300 words)

Objective: To summarise current quantitative evidence about differential use of primary care services amongst children and young people (CYP) in the UK, associations between the characteristics of CYP or their caregivers and service use, and evidence of horizontal inequity – unequal treatment for CYP patients with equal need.

Design: Systematic scoping review.

Setting: Primary care.

Eligibility criteria: English-language quantitative or mixed-methods studies published between 2012 and 2022.

Data sources: Medline, Embase, Scopus and Web of Science Social Sciences Citation Index, and Google Scholar.

Results: Twenty-two eligible studies were identified, covering general practice (n=14), dental health (n=4), child mental health services (n=3), and immunisation (n=1). Only eight studies (36%) controlled for variables associated with health need (e.g., age, birthweight, long-term conditions). In these, evidence of horizontal inequity in primary care use was reported for CYP living in deprived areas in England, with and without complex needs. Horizontal inequity was also identified in primary care mental health referrals for CYP in England identifying as mixed-race, Asian, or black ethnicity, compared to their White British peers. No evidence of horizontal inequity was observed, however, in primary care use for CYP in England exposed to parental depression, or for CYP children from low-income households in Scotland. Increasing CYP's age was associated with decreasing primary care use across included studies. No studies were found regarding CYP from Gypsy or Traveller communities, children in care, those with disabilities or special educational needs.

Conclusions: There is evidence that socio-economic factors impact upon CYP's primary care use, in particular age, ethnicity, and deprivation. However, better quality evidence is required to evaluate

For peer review only

horizontal inequity in CYP primary care use, and fill gaps in our knowledge of use for vulnerable CYP populations and the impact of policy and practice related 'supply side' of primary care.

Key messages

What is already known on this topic

- Key drivers for the recent growth in UK health inequity has primarily been assessed by research within the adult population.
- Whilst health disparities between CYP groups have been observed, little evidence is available for the relationship between the characteristics of CYP or their caregivers and disparities in primary care service use, taking into account underlying health needs.

What this study adds

- Few included studies took into account indicators of CYP's underlying health needs in their analysis, and thus could not assess horizontal inequity.
- Where health need was accounted for, primary care use reduced with CYP age or increasing deprivation. Some evidence for horizontal inequity in primary care mental health referrals and dental health was also reported.

How this study might affect research, practice or policy

- Our review summarises the current evidence base for known socio-economic drivers of inequity in CYP primary care use.
- Future research providing higher quality evidence, is required however, to further explore how their impact is affected by underlying health need, and to address the evidence gap found for vulnerable CYP populations.

Key words

Primary care; adolescents; children; access to healthcare; health equity; horizontal inequity; unmet need

For peer review only - http://bmjopen.bmj.com/site/about/guidelines.xhtml

Strengths and limitations (up to 5 bullet points)

- This review is the first to map evidence of horizontal inequity in primary care use among CYP in the UK. As such, it provides a summary of variation in primary care use and of the scale and type of inequalities in health care use amongst CYP, informing future research directions.
- Our review highlights a need for future quantitative studies of CYP primary care use to control for CYP's underlying health needs, in order to contribute to policy in England on reducing health inequalities.
- By restricting eligible publications to the last decade, findings are relevant to current health care systems and social context.
- We used systematic methods, including quality appraisal for robustness and reproducibility.
- As a scoping review, this review and its findings should not be treated as exhaustive.

For peer review only

For peer review only - http://bmjopen.bmj.com/site/about/guidelines.xhtml

Introduction

In recent years, health inequality (avoidable differences in health outcomes between groups) has been growing amongst the UK population (1,2). For children and young people (CYP) in England, those from Pakistani, Black African and Black Caribbean ethnic groups experience higher rates of infant mortality than other ethnicities (1). Vulnerable CYP (e.g., those with a learning disability or autism) have worse health and wellbeing outcomes, service experiences, poor outcomes associated with chronic conditions, and greater premature mortality risk (3–6). In recognition, the NHS England long term plan highlights the role of primary care in reducing health inequalities (7).

Horizontal inequity is defined as the unequal treatment for people with equal need (8). Despite evidence of horizontal inequity across the UK adult population (9,10), inequities amongst CYP is less well understood, particularly in primary care. This evidence gap is of particular concern given its potential to inhibit the effective development of policies to ensure that health care provision effectively meets CYP's level of health need.

Analysis of routinely collected national data, indicates that infants and preschool children have the highest general practice visit rates of any age group (11,12), whilst in the year 2021-22, nearly half of children aged 0 to 17 years in England receive NHS dental care annually (13). Beyond dental service access by patient age however, there are no government-provided national CYP statistics on the use of primary care use, or how their use varies for different sub-populations relative to health need.

In England, policies have been introduced over the last 30 years to specifically tackle deprivation-based inequity in general practice (14). Despite some evidence of improvements, their effectiveness may have been impaired by inadequate compensation for additional deprivation-related health care needs via the core general practice funding formula.

To effectively address equity within primary care and inform policy-making relating to the supply of care (e.g., service quality, quantity, and distribution) the best available evidence is needed for both

For peer review only - http://bmjopen.bmj.com/site/about/guidelines.xhtml

vulnerable and marginalised CYP groups, and the CYP population as a whole. In this article, we examine existing evidence on CYP primary care use and ask: what is the relationship between the characteristics of CYP or their caregivers and primary care service use in the UK, taking into account underlying health needs?

Method

The design of the review was informed by the Preferred Reporting Items for Systematic Reviews and Meta-Analysis extension for scoping reviews (PRISMA-ScR) guidance for rapid evidence reviews, developed by Tricco *et al*. (2018) (15). The protocol was registered in the Open Science Framework (Centre for Open Science, https://osf.io/mfc3z) and followed the PRISMA-ScR guide for the review design and reporting of methods and findings, and associated checklist (16). Due to the rapid nature of the review (10 weeks), the questions and search strategy were targeted to identify relevant articles that could be analysed within the review timeframe.

Inclusion/exclusion criteria

A study was included if it:

- Focused on the characteristics of CYP (aged 0 to 25 years), or families of children (aged 0 to 18 years);
- Was conducted on data for UK primary health care settings (general practice, community pharmacy, NHS provided dentistry);
- Outcomes were reported in terms of primary care use measures (e.g., primary care contacts, NHS dentist registration);
- Used quantitative or mixed-methods empirical methods;
- Was published between January 1st 2012 and 5th July 2022;

Studies were excluded if they:

For peer review only - http://bmjopen.bmj.com/site/about/guidelines.xhtml

- Focused on school-based health services (e.g., school nursing);

Search strategy

We used free-text and controlled terms (Appendix A) in four electronic databases Medline, Embase, Scopus and Web of Science (Social Sciences Citation Index), and screened the first 100 hits in Google Scholar.

Document selection

The search results were imported into Rayyan software (https://www.rayyan.ai/) for de-duplication, before being divided equally between two reviewers and screened independently by title and abstract. 10% of excluded articles for each reviewer were cross-checked by the other, to confirm agreement. As all excluded articles were agreed between reviewers at this stage, screening proceeded to a full text review by a single reviewer, with any articles for query referred to a second reviewer.

Data extraction and quality assessment

From included studies, two reviewers extracted: study sample/population; health care setting; area of health care; study design; methodology; variables controlling for health care need (where applicable); primary care contacts. We also extracted data for outpatient attendance, specialist referrals, and hospital-based service use (e.g., emergency presentations; hospital admissions) to explore potential associations between CYP primary care use and other health services. Study quality was assessed using the Mixed Methods Appraisal Tool (MMAT), (17,18). No study was excluded based on quality, but we report study quality.

For peer review only

Data synthesis

As a scoping review, assessment of study heterogeneity, and meta-analysis of study results were not performed. Study findings on CYP primary care service use and any associations with patient or caregiver characteristics, were narratively synthesised.

Patient involvement

As a scoping review of published literature, no patients were involved in the design, conduct or analysis of this research.

Results

We found 2,301 unique title/abstracts, of which 22 publications (reporting 21 studies) met the inclusion criteria (Figure 1)

Study characteristics

Study origin, health care setting and area of health

Most studies related to English primary care services (n=17); the rest were from Northern Ireland (n=2), Wales (n=1), Ireland (n=1) and Scotland (n=1), and UK (n=1) (see Table 1). Most focused upon utilisation of general practice services (n=14), with the remaining, dental services or oral health (n=4), child mental health services (n=3), and the uptake of immunisations (n=1).

Study designs and samples

Most of the eligible publications reported on cohort studies (n=17), with the remaining cross-sectional studies (n=5). The most common age ranges of CYP studied were 0 to 15 years (n=3), 0 to 18 years (n=3), 0 to 1 year (n=2), and 0 to 14 years (n=2) (for full details, see Table 1).

For peer review only - http://bmjopen.bmj.com/site/about/guidelines.xhtml

Characteristics studied

Eligible studies predominately investigated deprivation or social economic differences (n=16), sex or gender (n=13), ethnic group (n=11) or age (n=8) for potential associations with levels of primary care service use. Five of the included studies compared primary care use for CYP with a defined health condition: congenital abnormalities; CYP mental health; Down's syndrome; eczema (Table 1).

Fifteen of the included studies contained one or more health need variables in their analysis of CYP primary care use (Table 1). Eight studies controlled for health need in a general population sample, with CYP age (n=8), gestational age (n=3), and birthweight (n=2) the most often controlled for in regression analyses. Seven studies compared use between subsets of the primary CYP sample, the most commonly investigated subsets were CYP age (n=7), CYP with comorbidities (n=2), or CYP with defined conditions (n=2). None of the included studies accounted for variation in supply (e.g., CYP distance from practices, general practitioner [GP] appointment availability, GP to patient ratio) in the analysis of CYP use of primary care.

Outcomes

Table 2 summarises health care utilisation outcomes reported by included studies. The services most investigated were primary care attendance and/or GP consultations (n=13), outpatient attendances (n=7) and specialist referrals (n=5). Use of non-primary care services was recorded in nine of the eligible studies (see Table 2), to study their use in addition to primary care or to explore possible relationships between primary care presentations and the need for hospital-based care.

The relationship between CYP characteristics and use of publicly funded dental health services was investigated in four of the included studies. Outcomes reported for these studies focused upon the registration or rates of attendance with NHS (England) or General Dental Service (Northern Ireland) dental healthcare services, dental or oral health by examination, or publicly funded fee for service reimbursement for dental care provided (see Table 2).

For peer review only - http://bmjopen.bmj.com/site/about/guidelines.xhtml

Study quality

All studies bar one met four or five of the MMAT quality criteria (see Table 1). Fifteen of the twenty-two eligible publications included a statistical analysis and/or a study sample which accounted for health need. Of these, eight high quality publications controlled for health need in their analysis. The remaining seven publications were assigned high quality as their study designs were appropriate for their respective research questions, but we noted that indicators of health need as criteria were only used for deriving study samples (for full quality criteria, see Appendix B).

CYP or caregiver characteristics affecting primary care use

The next sections report the CYP or caregiver factors associated with UK primary care use. Although some characteristics were examined in multiple studies (for example, CYP age), or with large samples (for example, parental mental health), overall, the literature was fragmented with one or two studies reporting findings for some characteristics. Findings related to general practice are reported first in each section, followed by use of dental services. We have highlighted findings from high quality studies which have controlled for health need variables.

Sex or gender of CYP

Some evidence was apparent for gender-based differences in the use of primary care, and the routes by which CYP are referred to specialist services. Two high quality studies that controlled respectively for age, and age and type of mental health problem, found that males were more likely to be referred to child and adolescent mental health services (CAMHS) via education services than via primary care, compared to their female counterparts (19,20). Female CYP with a parental history of recurrent depression, were more likely to use primary care for the treatment of their own anxiety disorder, than their male counterparts (21). In contrast, in a study that characterised different patterns of CYP healthcare use in Northwest London, males were more likely to appear in the high

For peer review only - http://bmjopen.bmj.com/site/about/guidelines.xhtml

service use cluster compared to females, after adjusting for age, ethnicity, deprivation and long-term conditions (22).

Where dental services were studied, females were found to be more likely to use orthodontic services than males (23). Access to dental services also became differentiated with age with females being more likely to be registered with an NHS dentist than males by the age of 15-16 years, from no difference at 11-12 years of age (24).

Age of CYP

Consistent across included studies, increasing age of the child was associated with a fall in the use of primary care services, with older teenagers less likely to use primary care (12,22,24–26).

Deprivation or socio-economic classification

CYP from areas of higher deprivation in England were reported to have had lower levels of GP consultations, considering both CYP in the general population or CYP with congenital abnormalities (27,28). This was despite the overall need for health care being higher, reflected in a shift from the routine use of scheduled care services (e.g., GP consultations and outpatient attendance) to those that deal with unscheduled presentations (e.g., ED attendance), or hospital admissions. Exposure to deprivation was also observed to affect the uptake and timeliness of child vaccinations, with lower rates of uptake and later vaccinations for children from areas of highest deprivation (29). Of note, family GP practices with highest reported accessibility according to the GP Patient Survey were more likely to be located in more affluent areas, less likely to be in urban areas, and also had a lower proportion of their registered patient list being children (30).

In contrast to the above, the study of different patterns of health care use in Northwest London found higher levels of deprivation in clusters of CYP that had higher health service utilisation, including primary care (22). This finding indicates that a minority of CYP from the most deprived areas may be frequent attenders of primary care, but in general, more CYP from deprived areas

For peer review only

For peer review only - http://bmjopen.bmj.com/site/about/guidelines.xhtml

would be expected to have higher rates of primary care service use due to higher levels of health need. It should also be considered that the Beaney *et al*. (2021) study may not be nationally representative of the UK CYP population, due to the study population being drawn from an area with higher ethnic diversity and levels of deprivation, relative to national averages (22).

One high quality study that accounted for CYP age, birthweight, and parental assessment of CYP health among other factors, explored family income as a possible factor in inequality of GP service utilisation for samples of children from Scotland and the Republic of Ireland (31). We report only on the UK-related findings. The authors identified little or no overall income-related inequity in GP care for Scottish children in the overall analysis. The probability of GP visits was higher in younger children (2-year-olds) from less deprived backgrounds, although this was almost totally negated after adjustment for health and non-need determinants.

In dental care, children in the lowest socio-economic status group were more likely to require reparative dental treatments, and dental treatment overall, but were less likely to have consumed orthodontic services (24,32).

For children with congenital abnormalities, higher maternal education was associated with lower levels of primary care consultations and hospital service use - potentially a consequence of less educated parents requiring additional help from medical professionals to manage CYP complex needs (28). In contrast, lower educational attainment of the household reference person was associated with CYP having less time registered with an NHS dentist (24), and where the reference person had no qualifications, more than twice was spent on endodontics for the CYP than where a degree or greater was obtained (23).

Ethnic background

Associations were identified between ethnic background and primary care use, which were moderated by intersectionality. Three high quality studies, including one that controlled for age,

For peer review only - http://bmjopen.bmj.com/site/about/guidelines.xhtml

birthweight and gestational age (Hasan & West, 2021), reported that children from Black, Pakistani, Asian or Asian British ethnicity were observed to have GP consultation or prescription rates at higher levels than the majority population, while outpatient attendance and other services were relatively underutilised (22,27,33).

Babies born at term with low birth weights are recognised as having increased morbidity and mortality though infancy to adulthood (34). Some ethnic minorities (Indian, Bangladeshi, Pakistani, Black Caribbean, or Other) are observed to be overrepresented in terms of babies delivered at low birthweights (1). However, one high quality study that accounted for CYP gestational age, found that children of Pakistani ethnicity had greater primary care use than White British children irrespective of birthweight (35).

Two high quality studies that controlled respectively for age and type of mental health problem, found that children of Black, Asian, other (non-White), White other, or of mixed race, were more likely to be referred to child mental health services through routes other than primary care (e.g., education services, social services, youth justice), relative to their White British counterparts (19,20). Children of Asian, Black, White other, or other ethnicities were also less likely to have their case closed through non-attendance – a finding thought to be related to the higher rate of compulsory child mental health referrals (19,20).

Dental services reflected poorer outcomes in children of ethnic minorities, with those of White European, Bangladeshi or Pakistani ethnicity more likely to have poor dental health (tooth decay, teeth missing due to caries, fillings score, or greater numbers of untreated carious teeth), relative to children identifying as White British (36).

CYP with existing conditions

Differences in primary care service use were observed for children with existing conditions. One high quality study that controlled for multimorbidity found that CYP of Pakistani or 'Other' ethnic origin

For peer review only - http://bmjopen.bmj.com/site/about/guidelines.xhtml

with congenital abnormalities were found to have higher primary care use compared to their White British peers (28). A greater chance of eczema treatment escalation was also seen for CYP of Asian, Black African/Caribbean, Mixed or Other ethnicities, relative to those of White ethnicity (37). It is possible that these variations may be reflective of: condition-specific differences for different population subgroups; the performance of treatment pathways; GP-patient relationships; parental attitudes; or their presentation thresholds for primary care attendance. For children with Down's syndrome (DS), higher rates of primary care use (GP consultations, prescriptions) due to respiratory tract infections (RTIs) were observed, relative to a matched control group without DS (38). The authors noted that this may at least in part be due to increased RTI susceptibility and severity in children with DS, arising from vulnerabilities caused by differences in immunology, and airway morphology and function.

Parental MH

Two high quality studies, one of which accounted for CYP's age and presence of chronic conditions, found that CYP exposure to maternal mental illness was found to be more prevalent with increased deprivation, and associated with increased CYP primary health care use (25,39). Maternal mental illness exposed CYP had higher rates of primary care service contacts, outpatient attendance and specialist referrals. They also received more drug prescriptions for mental and behavioural disorders, physical, acute, or chronic diseases. Across all CYP who accessed primary care services, use was greater for those with mothers who had poor mental health than those without, which may indicate differences in health seeking behaviour or a higher level of health need (25,39).

In contrast, Potter *et al*. (2012) did not find any effect of having parents with current mental health conditions on the use of primary care by CYP (21). It should be noted however, that this study was based upon a relatively small sample size (n=333), compared to those used in Hope *et al*. (2021) and Dreyer *et al*. (2018) (n=489,255 and n=25,252 respectively) (25,39).

For peer review only - http://bmjopen.bmj.com/site/about/guidelines.xhtml

Migrant status

Eligible studies for primary care access and use by children with migrant backgrounds was limited. One high quality study that controlled for CYP age, reported that children of Indian ethnicity with a migrant background, were less likely to self-refer for mental health issues, although no differences in overall use of mental health services was observed (40).

Refugee status

Only one study (of lower quality) was identified on primary care use by a sample of children with refugee status of varied ethnicities (Afghanistan, Ethiopia, Gambia, Iran, Iraq, East Africa, Kenya, Nigeria, Pakistan, Somalia, Sudan, Tunisia, Vietnam, Zambia, Zimbabwe). No difference was found for medicines consumption relative to the control group studied (41), although evidence did indicate that over the counter, rather than prescribed medicines were more likely to be the primary source for refugee children.

Discussion

While the review identified multiple studies of CYP primary care use, relatively few took into account indicators of CYP's underlying health needs. Studies consistently found lower rates of CYP primary care use with increasing CYP age. There was evidence that CYP living in deprived areas in England used primary care less than their counterparts living in wealthier areas, a finding identified by both studies that accounted for indicators of underlying health needs and those that compared subsets of CYP. There was some evidence to suggest that CYP in deprived areas were more likely to use acute care services. Recent research has identified key principles to reduce the likelihood of health inequalities resulting from general practice, including accounting for differences within patient groups (42).

For peer review only

Included studies also suggested horizontal inequity in referrals to mental health services. Primary care services were less likely to refer male or non-White British CYP to CAMHS, potentially as a result of differences in help-seeking preferences, patient engagement, or 'GP gatekeeping' (43), highlighting an issue for which service and/or patient-focused solutions are being sought (44–46). Where NHS dental services were studied, a need for greater oral care education and outreach was indicated by the poor dental health and service access for CYP of non-White British ethnicity, and those from low socio-economic status and poorly educated households (24,32,36).

Two thirds of the studies (n=14) did not control for underlying health need in their analyses, and as a consequence it is difficult to understand whether differences in primary health care use reflect service inequalities or variation that is appropriately based on differences in need. Where studies did account for health indicators, age, gestational age and birthweight were the most commonly controlled for variables. Morbidity or CYP health status were largely absent from analyses. With regards other factors that may influence CYP primary care use, little reference was made to supply-side issues in general practice (e.g., appointment availability, practice location, GP to patient ratio), outside of a single study focused on accessibility via responses to the GP Patient Survey. Whilst the growing pressures on general practice are well reported (47), how these affect the CYP population and specifically how they may drive inequities in service use remains unclear.

Key gaps in the knowledge landscape were also identified. It should be noted that all included studies focused on CYP registered with primary care providers, with no studies found for unregistered patients, or service use in other care settings (e.g., walk-in centres), pointing toward a paucity in data collection in these areas. Little quantitative evidence was found regarding CYP primary care use in refugee or migrant populations, whilst no studies investigated primary care use for CYP from Gypsy or Traveller communities, care-experienced CYP, or for CYP with learning disabilities, autism or special educational needs.

For peer review only

This review was strengthened through clearly defined scoping review protocols, thus optimising search, screening and data extraction processes. By confining eligible publications to those from within the last decade, this study focuses on evidence relevant to current social dynamics and clinical practice. The review was however restricted by resource and time limitations, and thus the findings should not be treated as exhaustive.

Conclusion

Recent evidence suggests that CYP age, deprivation, ethnicity, and gender influence CYP primary care use, including NHS dental services. Studies that have accounted for indicators of health need, suggest that CYP living in deprived areas in England use primary care services less than CYP in wealthier areas with equal levels of need and have greater use of acute care. However, better quality evidence is required to adequately evaluate horizontal inequity in CYP primary care use and fill gaps in our knowledge of primary care use for vulnerable CYP populations, as well the impact of ‘supply side’ policy and practice for the delivery of high-quality primary care services to all CYP.

For peer review only - http://bmjopen.bmj.com/site/about/guidelines.xhtml

References

1. Public Health England. Local action on health inequalities. Understanding and reducing ethnic inequalities in health. 2018.

2. Marmot M, Allen J, Boyce T, Goldblatt P, Morrison J. Health equity in England: The Marmot review 10 years on. 2020.

3. Glover G, Williams R, Branford D, Avery R, Chauhan U, Hoghton M, et al. Prescribing of psychotropic drugs to people with learning disabilities and/or autism by general practitioners in England. 2015.

4. Crane L, Chester JW, Goddard L, Henry LA, Hill E. Experiences of autism diagnosis: A survey of over 1000 parents in the United Kingdom. Autism. 2015 Mar 25;20(2):153–62.

5. Department of Health and Social Care. The Government response to the Learning Disabilities Mortality Review (LeDeR) Programme Second Annual Report. 2016.

6. Wijlaars LPMM, Gilbert R, Hardelid P. Chronic conditions in children and young people: learning from administrative data. Arch Dis Child. 2016 Oct 1;101(10):881 LP – 885.

7. NHS England. The NHS Long Term Plan. 2019.

8. Pulok MH, van Gool K, Hajizadeh M, Allin S, Hall J. Measuring horizontal inequity in healthcare utilisation: a review of methodological developments and debates. Eur J Heal Econ. 2020;21(2):171–80.

9. Cookson R, Propper C, Asaria M, Raine R. Socio-Economic Inequalities in Health Care in England. Fisc Stud. 2016 Sep 1;37(3–4):371–403.

10. Turner AJ, Francetic I, Watkinson R, Gillibrand S, Sutton M. Socioeconomic inequality in access to timely and appropriate care in emergency departments. J Health Econ. 2022;85:102668.

11. Hobbs FDR, Bankhead C, Mukhtar T, Stevens S, Perera-Salazar R, Holt T, et al. Clinical workload in UK primary care: a retrospective analysis of 100 million consultations in England, 2007–14. Lancet. 2016;387(10035):2323–30.

12. Ruzangi J, Blair M, Cecil E, Greenfield G, Bottle A, Hargreaves D. Trends in healthcare use in children aged less than 15 years: A population-based cohort study in England from 2007 to 2017. BMJ Open. 2020;10(5):e033761.

13. NHS Digital. NHS Dental Statistics for England dashboard. 2022.

14. The Health Foundation. Tackling the inverse care law. Analysis of policies to improve general practice in deprived areas since 1990. 2022.

15. Tricco AC, Lillie E, Zarin W, O'Brien KK, Colquhoun H, Levac D, et al. PRISMA extension for scoping reviews (PRISMA-ScR): Checklist and explanation. Ann Intern Med. 2018;169(7):467–73.

16. Page MJ, McKenzie JE, Bossuyt PM, Boutron I, Hoffmann TC, Mulrow CD, et al. The PRISMA 2020 statement: an updated guideline for reporting systematic reviews. BMJ. 2021 Mar 29;372:n71.

17. Pace R, Pluye P, Bartlett G, Macaulay AC, Salsberg J, Jagosh J, et al. Testing the reliability and

For peer review only - http://bmjopen.bmj.com/site/about/guidelines.xhtml

efficiency of the pilot Mixed Methods Appraisal Tool (MMAT) for systematic mixed studies review. Int J Nurs Stud. 2012;49(1):47–53.

18. Souto RQ, Khanassov V, Hong QN, Bush PL, Vedel I, Pluye P. Systematic mixed studies reviews: Updating results on the reliability and efficiency of the mixed methods appraisal tool. Int J Nurs Stud. 2015;52(1):500–1.

19. Edbrooke-Childs J, Newman R, Fleming I, Deighton J, Wolpert M. The association between ethnicity and care pathway for children with emotional problems in routinely collected child and adolescent mental health services data. Eur Child Adolesc Psychiatry. 2016;25(5):539–46.

20. Edbrooke-Childs J, Patalay P. Ethnic Differences in Referral Routes to Youth Mental Health Services. J Am Acad Child Adolesc Psychiatry. 2019;58(3):368-375.e1.

21. Potter R, Mars B, Eyre O, Legge S, Ford T, Sellers R, et al. Missed opportunities: Mental disorder in children of parents with depression. Br J Gen Pract. 2012;62(600):e487–93.

22. Beaney T, Clarke J, Woodcock T, McCarthy R, Saravanakumar K, Barahona M, et al. Patterns of healthcare utilisation in children and young people: A retrospective cohort study using routinely collected healthcare data in Northwest London. BMJ Open. 2021;11(12).

23. Telford C, Murray L, Donaldson M, O'Neill C. An analysis examining socio-economic variations in the provision of NHS general dental practitioner care under a fee for service contract among adolescents: Northern Ireland Longitudinal Study. Community Dent Oral Epidemiol. 2012;40(1):70–9.

24. Telford C, O'Neill C. Changes in dental health investment across the adolescent years. Br Dent J. 2012;212(9).

25. Hope H, Osam C, Kontopantelis E, Hughes S, Munford L, Ashcroft D, et al. The healthcare resource impact of maternal mental illness on children and adolescents: UK retrospective cohort study. Br J Psychiatry. 2021;219(3):515–22.

26. Manikam L, Cupp M, Schilder A, Hayward A, Littlejohns P, Alexander E. Respiratory tract infection related healthcare utilisation in UK children with down's syndrome. Am J Respir Crit Care Med. 2018;197(MeetingAbstracts).

27. Coughlan C, Ruzangi J, Neale F, Nezafat Maldonado B, Blair M, Bottle A, et al. Social and ethnic group differences in healthcare use by children aged 0-14 years: A population-based cohort study in England from 2007 to 2017. Arch Dis Child. 2022;107(1):32–9.

28. Bishop C, Small N, Parslow R. Healthcare use for children with complex needs: Using routine health data linked to a multiethnic, ongoing birth cohort. BMJ Open. 2018;8(3):e018419.

29. Perry M, McGowan A, Roberts R, Cottrell S. Timeliness and equity of infant pertussis vaccination in wales: Analysis of the three dose primary course. Vaccine. 2020;38(6):1402–7.

30. Cecil E, Bottle A, Cowling TE, Majeed A, Wolfe I, Saxena S. Primary care access, emergency department visits, and unplanned short hospitalizations in the UK. Pediatrics. 2016;137(2).

31. Layte R, Nolan A. Income-related inequity in the use of GP services by children: a comparison of Ireland and Scotland. Eur J Health Econ. 2015;16(5):489–506.

32. Salomon-Ibarra C, Ravaghi V, Hill K, Jones C, Landes D, Morris A. Low rates of dental attendance by the age of one and inequality between local government administrative areas

For peer review only - http://bmjopen.bmj.com/site/about/guidelines.xhtml

in England. Community Dent Health. 2019;36(1):22–6.

33. Hasan T, West J. Body mass index and use and costs of primary care services among white british and pakistani children: Findings from the born in bradford cohort study. Arch Dis Child. 2021;106(SUPPL 1):A393–4.

34. Watkins WJ, Kotecha SJ, Kotecha S. All-Cause Mortality of Low Birthweight Infants in Infancy, Childhood, and Adolescence: Population Study of England and Wales. PLOS Med. 2016 May 10;13(5):e1002018.

35. West J, Kelly B, Collings P, Santorelli G, Mason D. Is small size at birth associated with early childhood morbidity in white British and Pakistani origin UK children aged 0-3? Findings from the born in Bradford cohort study. BMC Pediatr. 2018;18(1):22.

36. Marcenes W, Muirhead VE, Murray S, Redshaw P, Bennett U, Wright D. Ethnic disparities in the oral health of three- to four-year-old children in East London. Br Dent J. 2013;215(2).

37. de Lusignan S, Alexander H, Broderick C, Dennis J, McGovern A, Feeney C, et al. Patterns and trends in eczema management in UK primary care (2009-2018): A population-based cohort study. Clin Exp Allergy. 2021;51(3):483–94.

38. Manikam L, Schilder A, Lakhanpaul M, Littlejohns P, Alexander E. Respiratory tract infection-related healthcare utilisation in children with Down's syndrome. Infection. 2020;48(3):403–10.

39. Dreyer K, Williamson R, Hargreaves D, Rosen R. Associations between parental mental health and other family factors and healthcare utilisation among children and young people: A retrospective, cross-sectional study of linked healthcare data. BMJ Paediatr Open. 2018;2(1):e000266.

40. Vostanis P, Svirydzenka N, Dugard P, Singh S, Dogra N. Mental health service use by adolescents of Indian and White origin. Arch Dis Child. 2013 Oct 1;98(10):764 LP – 767.

41. Alkahtani S, Cherrill J, Millward C, Grayson K, Hilliam R, Sammons H, et al. Access to medicines by child refugees in the east midlands region of england: A cross-sectional study. BMJ Open. 2014;4(12).

42. Gkiouleka A, Wong G, Sowden S, Bambra C, Siersbaek R, Manji S, et al. Reducing health inequalities through general practice. Lancet Public Heal. 2023 Jun 1;8(6):e463–72.

43. Sripa P, Hayhoe B, Garg P, Majeed A, Greenfield G. Impact of GP gatekeeping on quality of care, and health outcomes, use, and expenditure: a systematic review. Br J Gen Pract. 2019;69(682):E294–303.

44. Greater Manchester Poverty Action. Greater Manchester Poverty Monitor 2022 [Internet]. 2022 [cited 2023 Apr 5]. Available from: https://www.gmpovertyaction.org/poverty-monitor-2022/

45. The King's Fund. The NHS's role in tackling poverty: Awareness, action and advocacy. 2021.

46. The King's Fund. Poverty and the health and care system: The role of data and partnership in bringing change. 2022.

47. The King's Fund. Understanding pressures in general practice. 2016.

48. Markkula N, Cabieses B, Lehti V, Uphoff E, Astorga S, Stutzin F. Use of health services among

For peer review only - http://bmjopen.bmj.com/site/about/guidelines.xhtml

international migrant children - a systematic review. Global Health. 2018;14(1).

For peer review only

For peer review only - http://bmjopen.bmj.com/site/about/guidelines.xhtml

*Table 1: Characteristics of included studies (Health need indicator variables for the characteristics of CYP and their caregivers in **bold**).*

**Author (year)** ***sample location***	**Design**	**Health care setting; primary focus of study**	**Sample population**	**Age of sample**	**CYP/caregiver characteristics examined**		**MMAT Score**
					**Sample subset(s)**	**Controlling variable(s)**	
Coughlan *et al*. (2022) *England*	Retrospective cohort study	*General practice, outpatients, emergency department, hospital admissions*: Differences in health care use by health care setting, level of deprivation and ethnic group.	1,484,455 (from 408 GP practices)	0 – 14 years	Ethnicity, deprivation (IMD)	-	****
Beaney *et al*. (2021) *England*	Retrospective cohort study	*General practice, outpatients, emergency department, hospital admissions*: Patterns of healthcare utilisation in the CYP population in Northwest London	378,309	0 – 15 years	**Age**, sex, ethnicity, deprivation (IMD)	-	***** §
de Lusignan *et al*. (2021) *England*	Population-based cohort study	*General practice, specialist referrals:* Use of services and treatment for eczema patients	53,780	0 – 18 years	**Age**, sex, ethnicity, deprivation (IMD), rural-urban classification	-	***** §
Hasan & West (2021) *England*	Prospective cohort study	*Primary care consultations (not specified):* Effects of obesity and ethnicity	7,815 (3,469 White British, 4,346 Pakistani)	8 years	Ethnicity	**Age**, sex, ethnicity, deprivation (not specified), **birthweight**, **gestational age**, maternal BMI, maternal age	*****
Hope *et al*. (2021) *England*	Retrospective cohort study	*General practice, outpatients, emergency department, hospital admissions*: Children exposed to maternal mental illness	489,255	0 – 17 years	**Age**, Ethnicity	Deprivation (IMD)	***** §
Manikam *et al*. (2020) *England*	Retrospective cohort study	*General practice, hospital admissions*: Respiratory tract infection-related healthcare utilisation in children with Down's syndrome	5,866 (992, Down's syndrome, 4,874 control)	0 – 18 years	**Age**, sex, **comorbidities**, **respiratory tract infection type**	-	***** §
Perry *et al*. (2020) *Wales*	Routine health data linked to birth cohort	*General practice (vaccinations)*: Association of deprivation with pertussis vaccination uptake	163,733	0 – 1 years	Deprivation (Welsh IMD)	-	****
Ruzangi *et al*. (2020) *England*	Population-based cohort study	*General practice, outpatients, emergency department, hospital admissions*: Analysis of individual-level patient data to derive population-based estimates of healthcare utilisation	1,484,455 (from 408 GP practices)	0 – 14 years	**Age**	-	***** §
Edbrooke-Childs & Patalay (2019) *England*	Routine youth mental health services data	*General practice, emergency department, mental health services, child health services, education services*: Referral routes to youth mental health services	14,588	0 – 25 years (0-5, 6-12, 13-25 years)	-	**Age**, sex, ethnicity, deprivation (IDACI), **problem type**, contextual factors (home life, school, community, engagement)	*****
Salomon-Ibarra *et al*. (2019) *England*	Routine health data linked to birth cohort	*NHS England dental services:* Dental attendance	155,308	0 – 1 year	-	Deprivation (IMD)	****

For peer review only - http://bmjopen.bmj.com/site/about/guidelines.xhtml

Bishop *et al.* (2018) *England*	Prospective birth cohort study	*General practice, outpatients, emergency department, hospital admissions*: Children with congenital abnormalities on utilisation of primary and secondary care and referrals to specialists.	13,857	0 – 4 years	-	Deprivation (Means-tested benefit status), ethnicity, maternal age, maternal educational attainment, consanguinity, **multimorbidity**	*****
Dreyer *et al.* (2018) *England*	Retrospective, cross-sectional study	*General practice, outpatients, emergency department, hospital admissions*: Parental mental health and other family factors	25,252	0 – 15 years	-	**Age**, sex, deprivation (IMD), **CYP long-term conditions** Parent: age, healthcare utilisation, long-term conditions Household: Number of adults aged 18–55 years, number of CYP aged 0–15 years, presence of any patient aged ≥65 years, total number of patients in household	*****
West *et al.* (2018) *England*	Prospective cohort study	*General practice, outpatients, emergency department, hospital admissions*: Early childhood morbidity and small size at birth	8,850	0 – 3 years	Ethnicity	Sex, maternal parity, **gestational age**, maternal age, social economic information (maternal education, housing tenure, means-tested bene-fits), smoking	*****
Cecil *et al.* (2016) *England*	Cross-sectional, population- based study	*General practice, emergency department, hospital admissions*: Primary care access, emergency department visits, and unplanned short hospitalizations	9.5 million	0 – 15 years	-	Practice deprivation (IMD), urban/rural profile, percentage of the registered population aged ≥65 years	****
Edbrooke-Childs *et al.* (2016) *England*	Routinely collected child and adolescent mental health services data	*General practice, emergency department, social services, education services, child health services, youth justice, self-referral, other services:* Children with mental health problems	11,592 (26 CAMHS)	0 – 18 years	-	**Age**, sex, ethnicity	*****
Layte & Nolan (2015) *Ireland and Scotland*	Child cohort studies	*General practice:* Use of GP services	9,719	2 and 4 years	-	**Age**, sex, deprivation (family income) Health need: **Birthweight, gestation, parental assessment of child's general health status, exposure to accidents** Non-need: Number of siblings, maternal highest level of education, maternal employment status, household composition, maternal ethnicity Health care entitlements	*****
Alkahtani *et al.* (2014) *England*	Cross-sectional study	*General practice, over the counter medication:* Access to medicines by child refugees	216 (117 refugee, 99 control)	4 – 5 years	-	-	***

For peer review only - http://bmjopen.bmj.com/site/about/guidelines.xhtml

Vostanis *et al.* (2013) *England* †	Cross-sectional study	*General practice, CAMHS and paediatric hospital services*: Difference in level of need and service utilisation by young ethnic minority groups.	2,900	13 – 15 years	-	**Age**, ethnicity, free school meals, abnormal score (SDQ or MFQ-short), who the child lives with	*****
Marcenes *et al.* (2013) *England*	Cluster sampled, cross-sectional study	*Dental health (British Association for the Study of Community Dentistry):* Oral health	1,285	3 – 4 years	Deprivation (IMD)	Sex, ethnicity, London borough	****
Potter *et al.* (2012) *UK*	Longitudinal cohort study	General practice, paediatrics, mental health services, social services, youth justice: Mental disorder in children of parents with depression	333	9 – 17 years	**Age**, sex, parental age, family status, parental problem drinking, **child comorbidity (≥2 disorders)**, **child suicidality/ self-harm**, lack of parental concern, parental DSM-IV current depressive episode, frequent parental depressive episodes (≥4)	-	**** §
Telford & O'Neill (2012) *Northern Ireland*	Longitudinal cohort study	*General Dental Service (Northern Ireland):* Dental health investment	13,564	11 – 12 years	**Age**	Sex, deprivation (NS-SEC), highest educational attainment, parental marital status, number of siblings	***** §
Telford *et al.* (2012) *Northern Ireland*	Longitudinal cohort study	*General Dental Service (Northern Ireland):* Provision of NHS general dental practitioner care	12,846	11 – 12 years	-	Highest educational attainment, parental marital status, number of siblings, orthodontic treatment	****

† Identified from Markkula *et al.*, 2018.

‡ MMAT score: ***** high quality; **** high quality; *** average quality; ** low quality; * low quality; No stars, poor quality.

§ Indicator of health need used to define study sample only.

Abbreviations: CAMHS, child and adolescent mental health services; CYP, children and young people; GP, general practice; IDACI, Income Deprivation Affecting Children Index; IMD, Index of Multiple Deprivation; MFQ-short, Mood and Feelings Questionnaire-short; MMAT, Mixed Methods Appraisal Tool; NHS, National Health Service; NS-SEC, National statistics socio-economic classification of occupation; SDQ, Strengths and Difficulties Questionnaire.

For peer review only

For peer review only - http://bmjopen.bmj.com/site/about/guidelines.xhtml

Table 2. Outcomes reported in included studies.

	Health services										Dental			
	Attendances/Consultations					Other								
Author (year)	Primary care (GP/other)	Outpatients	Emergency Department/ A&E	Hospital (Elective)	Specialist referrals	Medication (Prescription/OTC)	GP registration	Treatment escalation	Immunisation	Non-medical services*	Dental attendances	Dental registration	Dental/Oral health	Reimbursement
Coughlan *et al*. (2022)	■	■	■	■										
Beaney *et al*. (2021)	■	■	■	■										
de Lusignan *et al*. (2021)	■				■ †			■						
Hasan & West (2021)	■					■								
Hope *et al*. (2021)	■	■	■	■	■ ‡	■								
Manikam *et al*. (2020)	■		■	■		■								
Perry *et al*. (2020)									■					
Ruzangi *et al*. (2020)	■	■	■	■										
Edbrooke-Childs & Patalay (2019)					■ §									
Salomon-Ibarra *et al*. (2019)											■			
Bishop *et al*. (2018)	■	■	■	■	■									
Dreyer *et al*. (2018)	■	■	■	■										
West *et al*. (2018)	■	■	■	■		■								
Cecil *et al*. (2016)			■	■										
Edbrooke-Childs *et al*. (2016)					■ §									
Layte & Nolan (2015)	■													
Alkhatani *et al*. (2014)	■					■	■							
Vostanis *et al*. (2013)	■									■				
Marcenes *et al*. (2013)													■	
Potter *et al*. (2012)										■				
Telford & O'Neill (2012)												■		
Telford *et al*. (2012)												■		■

Abbreviations: GP, general practice.

* Schools and special educational needs services, social services, or youth justice services.

† Dermatologist, GP with a specialty interest in dermatology, or dermatology specialist nurse.

‡ Not specified.

§ Mental health services referrals.

For peer review only

Funding statement

This study is funded by the National Institute for Health and Social Care Research (NIHR) through the Children and Families Policy Research Unit (PR-PRU-1217-21301). The views expressed are those of the authors and not necessarily those of the NIHR or the Department of Health and Social Care.

Ethics approval

As a scoping review, this research did not involve participants and thus ethics approval was not required.

Competing interests statement

The authors of this paper declare no conflicts of interest.

For peer review only - http://bmjopen.bmj.com/site/about/guidelines.xhtml

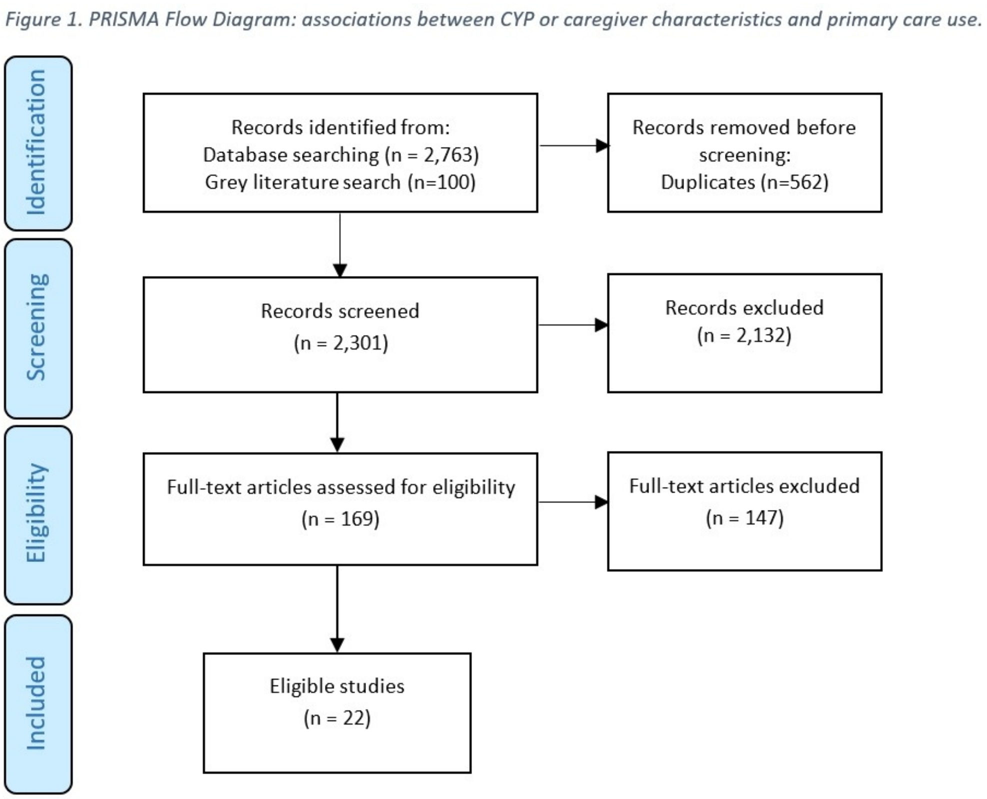

Figure 1. PRISMA Flow Diagram: associations between CYP or caregiver characteristics and primary care use.

242x194mm (96 x 96 DPI)

Patient and caregiver characteristics associated with differential use of primary care for children and young people in the UK: a scoping review

Appendices

Appendix A: Search terms and results

Ovid

- Embase 1996 to 2022 Week 24
- Ovid MEDLINE(R) 1996 to June Week 2 2022

Search conducted 23rd June 2022

	Search	Terms	Hits
**1**	Service access	exp "Health Services Accessibility"/ or "access to health services".tw. or "access to care".tw. or exp "Health Equity"/ or "health equity".tw. or exp "Health Literacy"/ or "health literacy".tw. or "healthcare use".tw. or "healthcare utilisation".tw. or "healthcare utilization".tw. or inequality.tw. or inequalities.tw. or equality.tw. or disparity.tw. or disparities.tw. or unequal.tw. or gap*.tw. or gradients.tw. or disadvantage.tw.	1,076,198
**2**	Primary care	exp "Primary Health Care"/ or "primary health care".tw. or "primary care".tw. or exp "Primary Care Nursing"/ or exp "Physicians, Primary Care"/ or exp "General Practice"/ or "general practice*".tw. or "GP surger*".tw. or exp "General Practice, Dental"/ or dentist*.tw. or exp "General Practitioners"/ or "general practitioner*".tw. or exp "Community Pharmacy Services"/ or "community pharmac*".tw.	766,436
**3**	CYP	exp Child/ or child*.tw. or exp Adolescent/ or adolescen*.tw. or exp Infant/ or infant*.tw. or "young people".tw. or youth.tw. or juvenile*.tw. or teenager*.tw. or exp "Parent-Child Relations"/ or "young adult*".tw. or paediatri*.tw.	6,208,476
**4**	UK	("United Kingdom" or England or Wales or Scotland or "Northern Ireland" or "N. Ireland").af.	13,100,534
**5**	Quantitiative studies	(quantit* or consultation* or episode* or visit* or longitudin* or patient-level).af.	4,071,128
**6**		1 and 2 and 3 and 4 and 5	1,932
**7**	Limit to human studies	limit 6 to humans	1,915
**8**	Limit to years 2010 - 2022	limit 7 to yr="2010 - 2022"	1,427
**9**	Deduplication<br><br>Preferences:	remove duplicates from 8<br><br>Deduplication (Ovid)<br>Has full text; Embase > Ovid MEDLINE(R)	1,097

For peer review only - http://bmjopen.bmj.com/site/about/guidelines.xhtml

Scopus

Search conducted 23rd June 2022

	Search	Terms	Hits
**1**	Service access	INDEXTERMS("Health Services Accessibility") OR TITLE-ABS("access to health services") OR TITLE-ABS("access to care") OR INDEXTERMS("Health Equity") OR TITLE-ABS("health equity") OR INDEXTERMS("Health Literacy") OR TITLE-ABS("health literacy") OR TITLE-ABS("healthcare use") OR TITLE-ABS("healthcare utilisation") OR TITLE-ABS("healthcare utilization") OR TITLE-ABS(inequality) OR TITLE-ABS(inequalities) OR TITLE-ABS(equality) OR TITLE-ABS(disparity) OR TITLE-ABS(disparities) OR TITLE-ABS(unequal) OR TITLE-ABS(gap*) OR TITLE-ABS(gradients) OR TITLE-ABS(disadvantage)	2,671,314
**2**	Primary care	INDEXTERMS("Primary Health Care") OR TITLE-ABS("primary health care") OR TITLE-ABS("primary care") OR INDEXTERMS("Primary Care Nursing") OR INDEXTERMS("Physicians, Primary Care") OR INDEXTERMS("General Practice") OR TITLE-ABS("general practice*") OR TITLE-ABS("GP surger*") OR INDEXTERMS("General Practice, Dental") OR TITLE-ABS(dentist*) OR INDEXTERMS("General Practitioners") OR TITLE-ABS("general practitioner*") OR INDEXTERMS("Community Pharmacy Services") OR TITLE-ABS("community pharmac*")	481,759
**3**	CYP	INDEXTERMS(Child) OR TITLE-ABS(child*) OR INDEXTERMS(Adolescent) OR TITLE-ABS(adolescen*) OR INDEXTERMS(Infant) OR TITLE-ABS(infant*) OR TITLE-ABS("young people") OR TITLE-ABS(youth) OR TITLE-ABS(juvenile*) OR TITLE-ABS(teenager*) OR INDEXTERMS("Parent-Child Relations") OR TITLE-ABS("young adult*") OR TITLE-ABS(paediatri*)	5,664,633
**4**	UK	ALL("United Kingdom") OR ALL(England) OR ALL(Wales) OR ALL(Scotland) OR ALL("Northern Ireland") OR ALL("N. Ireland")	12,674,770
**5**	Quantitiative studies	ALL(quantit* OR consultation* OR episode* OR visit* OR longitudin* OR patient-level)	11,926,797
**6**	Combine 1-5	#1 AND #2 AND #3 AND #4 AND #5	2,018
**7**	Limit to articles or reviews	LIMIT-TO (DOCTYPE, "ar") OR LIMIT-TO (DOCTYPE, "re")	1,958
**8**	Limit to years 2010 - 2022	LIMIT-TO (PUBYEAR, 2022) OR LIMIT-TO (PUBYEAR, 2021) OR LIMIT-TO (PUBYEAR, 2020) OR LIMIT-TO (PUBYEAR, 2019) OR LIMIT-TO (PUBYEAR, 2018) OR LIMIT-TO (PUBYEAR, 2017) OR LIMIT-TO (PUBYEAR, 2016) OR LIMIT-TO (PUBYEAR, 2015) OR LIMIT-TO (PUBYEAR, 2014) OR LIMIT-TO (PUBYEAR, 2013) OR LIMIT-TO (PUBYEAR, 2012) OR LIMIT-TO (PUBYEAR, 2011) OR LIMIT-TO (PUBYEAR, 2010)	1,372
**9**	Limit to English language publications	LIMIT-TO (LANGUAGE,"English")	1,354

For peer review only - http://bmjopen.bmj.com/site/about/guidelines.xhtml

Web of Science

Web of Science Core Collection: All editions

Search conducted 22nd June 2022

	Search	Terms	Hits
**1**	Service access	ALL=("Health Services Accessibility" OR "access to health services" OR "access to care" OR "Health Equity" OR "health equity" OR "Health Literacy" OR "health literacy" OR "healthcare use" OR "healthcare utilisation" OR "healthcare utilization" OR inequality OR inequalities OR equality OR disparity OR disparities OR unequal OR gap* OR gradients OR disadvantage)	2,284,588
**2**	Primary care	ALL=("Primary Health Care" OR "primary health care" OR "primary care" OR "Primary Care Nursing" OR "Physicians, Primary Care" OR "General Practice" OR "general practice*" OR "GP surger*" OR "General Practice, Dental" OR dentist* OR "General Practitioners" OR "general practitioner*" OR "Community Pharmacy Services" OR "community pharmac*")	444,274
**3**	CYP	ALL=(Child OR child* OR Adolescent OR adolescen* OR Infant OR infant* OR "young people" OR youth OR juvenile* OR teenager* OR "Parent-Child Relations" OR "young adult*" OR paediatri*)	4,378,563
**4**	UK	ALL=("United Kingdom" OR England OR Wales OR Scotland OR "Northern Ireland" OR "N. Ireland")	6,796,242
**5**	Quantitiative studies	ALL=(quantit* OR consultation* OR episode* OR visit* OR longitudin* OR patient-level)	3,328,555
**6**		#1 AND #2 AND #3 AND #4 AND #5	408
**7**	Limit to years 2010 - 2022	#6<br>Publication date: 2010-01-01 to 2022-01-01	315
**8**	Limit to articles or reviews	#7 AND DT=(Article OR Review)	313
**9**	Limit to English language publications	(#8) AND LA=(English)	312

Google

Grey literature search

Search conducted 5th July 2022

Search terms

(site:.org.uk OR site:.gov.uk) AND General Practice* OR General Practitioner* OR Primary Care OR GP* AND Inequality OR Inequalities

For peer review only

Appendix B: Mixed Methods Appraisal Tool (MMAT) quality assessment of eligible studies

Criteria scoring: 0 = Not met; 1 = Partially met; 2 = Fully met.

Total score (Max 14): * ≤5; ** 6-9; *** 10-11; **** 12-13; ***** 14.

		Publication										
Category of study designs	Methodological quality criteria	Coughlan *et al.*, 2022	Beaney *et al.*, 2021	de Lusignan *et al.*, 2021	Hasan & West, 2021	Hope *et al.*, 2021	Manikam *et al.*, 2020	Perry *et al.*, 2020	Ruzangi *et al.*, 2020	Edbrooke-Childs & Patalay, 2019	Salomon-Ibarra *et al.*, 2019	Bishop *et al.*, 2018
Screening questions (for all types)	S1. Are there clear research questions?	2	2	2	2	2	2	2	2	2	2	2
	S2. Do the collected data allow to address the research questions?	2	2	2	2	2	2	2	2	2	2	2
Further appraisal may not be feasible or appropriate when the answer is ‘No’ or ‘Can’t tell’ to one or both screening questions.												
Quantitative non-randomized controlled trials	3.1. Are the participants representative of the target population?					2						2
	3.2. Are measurements appropriate regarding both the outcome and intervention (or exposure)?					2						2
	3.3. Are there complete outcome data?					2						2
	3.4. Are the confounders accounted for in the design and analysis?					2						2
	3.5. During the study period, is the intervention administered (or exposure occurred) as intended?					2						2
Quantitative descriptive	4.1. Is the sampling strategy relevant to address the research question?	2	2	2	2		2	2	2	2	2	
	4.2. Is the sample representative of the target population?	2	2	2	2		2	2	2	2	2	
	4.3. Are the measurements appropriate?	2	2	2	2		2	2	2	2	2	
	4.4. Is the risk of nonresponse bias low?	2	2	2	2		2	2	2	2	2	
	4.5. Is the statistical analysis appropriate to answer the research question?	2	2	2	2		2	2	2	2	2	
Total score		14	14	14	14	14	14	14	14	14	14	14

For peer review only - http://bmjopen.bmj.com/site/about/guidelines.xhtml

		Publication										
Category of study designs	Methodological quality criteria	Dreyer *et al.*, 2018	Vostanis et al., 2013 †	West *et al.*, 2018	Cecil *et al.*, 2016	Edbrooke-Childs *et al.*, 2016	Layte & Nolan, 2015	Alkahtani *et al.*, 2014	Marcenes *et al.*, 2013	Potter *et al.*, 2012	Telford & O'Neill, 2012	(C. Telford *et al.*, 2012)
Screening questions (for all types)	S1. Are there clear research questions?	2	2	2	2	2	2	2	2	2	2	2
	S2. Do the collected data allow to address the research questions?	2	2	2	2	2	2	2	2	2	2	2
Further appraisal may not be feasible or appropriate when the answer is 'No' or 'Can't tell' to one or both screening questions.												
Quantitative non-randomized controlled trials	3.1. Are the participants representative of the target population?				2		2	1				
	3.2. Are measurements appropriate regarding both the outcome and intervention (or exposure)?				2		2	2				
	3.3. Are there complete outcome data?				2		2	2				
	3.4. Are the confounders accounted for in the design and analysis?				2		2	0				
	3.5. During the study period, is the intervention administered (or exposure occurred) as intended?				2		2	2				
Quantitative descriptive	4.1. Is the sampling strategy relevant to address the research question?	2	2	2		2			2	2	2	2
	4.2. Is the sample representative of the target population?	2	2	2		2			2	1	2	2
	4.3. Are the measurements appropriate?	2	2	2		2			2	2	2	2
	4.4. Is the risk of nonresponse bias low?	2	2	2		2			2	2	2	2
	4.5. Is the statistical analysis appropriate to answer the research question?	2	2	2		2			2	2	2	2
Total score		14	14	14	14	14	14	11	14	13	14	14

† Identified from Markkula *et al.*, 2018.

For peer review only

References

Alkahtani, S., Cherrill, J., Millward, C., Grayson, K., Hilliam, R., Sammons, H., & Choonara, I. (2014). Access to medicines by child refugees in the east midlands region of england: A cross-sectional study. *BMJ Open*, *4*(12). https://doi.org/10.1136/bmjopen-2014-006421

Beaney, T., Clarke, J., Woodcock, T., McCarthy, R., Saravanakumar, K., Barahona, M., Blair, M., & Hargreaves, D. S. (2021). Patterns of healthcare utilisation in children and young people: A retrospective cohort study using routinely collected healthcare data in Northwest London. *BMJ Open*, *11*(12). https://doi.org/10.1136/bmjopen-2021-050847

Bishop, C., Small, N., & Parslow, R. (2018). Healthcare use for children with complex needs: Using routine health data linked to a multiethnic, ongoing birth cohort. *BMJ Open*, *8*(3), e018419. https://doi.org/http://dx.doi.org/10.1136/bmjopen-2017-018419

Cecil, E., Bottle, A., Cowling, T. E., Majeed, A., Wolfe, I., & Saxena, S. (2016). Primary care access, emergency department visits, and unplanned short hospitalizations in the UK. *Pediatrics*, *137*(2). https://doi.org/10.1542/peds.2015-1492

Coughlan, C., Ruzangi, J., Neale, F., Nezafat Maldonado, B., Blair, M., Bottle, A., Saxena, S., & Hargreaves, D. (2022). Social and ethnic group differences in healthcare use by children aged 0-14 years: A population-based cohort study in England from 2007 to 2017. *Archives of Disease in Childhood*, *107*(1), 32–39. https://doi.org/https://dx.doi.org/10.1136/archdischild-2020-321045

de Lusignan, S., Alexander, H., Broderick, C., Dennis, J., McGovern, A., Feeney, C., & Flohr, C. (2021). Patterns and trends in eczema management in UK primary care (2009-2018): A population-based cohort study. *Clinical and Experimental Allergy*, *51*(3), 483–494. https://doi.org/http://dx.doi.org/10.1111/cea.13783

Dreyer, K., Williamson, R., Hargreaves, D., & Rosen, R. (2018). Associations between parental mental health and other family factors and healthcare utilisation among children and young people: A retrospective, cross-sectional study of linked healthcare data. *BMJ Paediatrics Open*, *2*(1), e000266. https://doi.org/https://dx.doi.org/10.1136/bmjpo-2018-000266

Edbrooke-Childs, J., Newman, R., Fleming, I., Deighton, J., & Wolpert, M. (2016). The association between ethnicity and care pathway for children with emotional problems in routinely collected child and adolescent mental health services data. *European Child and Adolescent Psychiatry*, *25*(5), 539–546. https://doi.org/10.1007/s00787-015-0767-4

Edbrooke-Childs, J., & Patalay, P. (2019). Ethnic Differences in Referral Routes to Youth Mental Health Services. *Journal of the American Academy of Child and Adolescent Psychiatry*, *58*(3), 368-375.e1. https://doi.org/https://dx.doi.org/10.1016/j.jaac.2018.07.906

Hasan, T., & West, J. (2021). Body mass index and use and costs of primary care services among white british and pakistani children: Findings from the born in bradford cohort study. *Archives of Disease in Childhood*, *106*(SUPPL 1), A393–A394. https://doi.org/http://dx.doi.org/10.1136/archdischild-2021-rcpch.683

Hope, H., Osam, C., Kontopantelis, E., Hughes, S., Munford, L., Ashcroft, D., & Pierce, M. (2021). The healthcare resource impact of maternal mental illness on children and adolescents: UK retrospective cohort study. *British Journal of Psychiatry*, *219*(3), 515–522. https://doi.org/https://dx.doi.org/10.1192/bjp.2021.65

Layte, R., & Nolan, A. (2015). Income-related inequity in the use of GP services by children: a comparison of Ireland and Scotland. *The European Journal of Health Economics : HEPAC : Health Economics in Prevention and Care*, *16*(5), 489–506.

For peer review only - http://bmjopen.bmj.com/site/about/guidelines.xhtml

https://doi.org/https://dx.doi.org/10.1007/s10198-014-0587-3

Manikam, L., Schilder, A., Lakhanpaul, M., Littlejohns, P., & Alexander, E. (2020). Respiratory tract infection-related healthcare utilisation in children with Down's syndrome. *Infection*, *48*(3), 403–410. https://doi.org/http://dx.doi.org/10.1007/s15010-020-01408-5

Marcenes, W., Muirhead, V. E., Murray, S., Redshaw, P., Bennett, U., & Wright, D. (2013). Ethnic disparities in the oral health of three- to four-year-old children in East London. *British Dental Journal*, *215*(2). https://doi.org/10.1038/sj.bdj.2013.687

Markkula, N., Cabieses, B., Lehti, V., Uphoff, E., Astorga, S., & Stutzin, F. (2018). Use of health services among international migrant children - a systematic review. *Globalization and Health*, *14*(1). https://doi.org/10.1186/s12992-018-0370-9

Perry, M., McGowan, A., Roberts, R., & Cottrell, S. (2020). Timeliness and equity of infant pertussis vaccination in wales: Analysis of the three dose primary course. *Vaccine*, *38*(6), 1402–1407. https://doi.org/https://dx.doi.org/10.1016/j.vaccine.2019.12.001

Potter, R., Mars, B., Eyre, O., Legge, S., Ford, T., Sellers, R., Craddock, N., Rice, F., Collishaw, S., Thapar, A., & Thapar, A. K. (2012). Missed opportunities: Mental disorder in children of parents with depression. *British Journal of General Practice*, *62*(600), e487–e493. https://doi.org/10.3399/bjgp12X652355

Ruzangi, J., Blair, M., Cecil, E., Greenfield, G., Bottle, A., & Hargreaves, D. (2020). Trends in healthcare use in children aged less than 15 years: A population-based cohort study in England from 2007 to 2017. *BMJ Open*, *10*(5), e033761. https://doi.org/http://dx.doi.org/10.1136/bmjopen-2019-033761

Salomon-Ibarra, C., Ravaghi, V., Hill, K., Jones, C., Landes, D., & Morris, A. (2019). Low rates of dental attendance by the age of one and inequality between local government administrative areas in England. *Community Dental Health*, *36*(1), 22–26. https://doi.org/10.1922/CDH_4390Salomon-Ibarra05

Telford, C. J., & O'Neill, C. (2012). Summary of: Changes in dental health investment across the adolescent years. *British Dental Journal*, *212*(9), 438–439. https://doi.org/10.1038/sj.bdj.2012.372

Telford, C., Murray, L., Donaldson, M., & O'Neill, C. (2012). An analysis examining socio-economic variations in the provision of NHS general dental practitioner care under a fee for service contract among adolescents: Northern Ireland Longitudinal Study. *Community Dentistry and Oral Epidemiology*, *40*(1), 70–79. https://doi.org/http://dx.doi.org/10.1111/j.1600-0528.2011.00649.x

Vostanis, P., Svirydzenka, N., Dugard, P., Singh, S., & Dogra, N. (2013). Mental health service use by adolescents of Indian and White origin. *Archives of Disease in Childhood*, *98*(10), 764 LP – 767. https://doi.org/10.1136/archdischild-2013-303772

West, J., Kelly, B., Collings, P., Santorelli, G., & Mason, D. (2018). Is small size at birth associated with early childhood morbidity in white British and Pakistani origin UK children aged 0-3? Findings from the born in Bradford cohort study. *BMC Pediatrics*, *18*(1), 22. https://doi.org/http://dx.doi.org/10.1186/s12887-018-0987-0

For peer review only - http://bmjopen.bmj.com/site/about/guidelines.xhtml

Reporting checklist for systematic review (with or without a meta-analysis).

Based on the PRISMA guidelines.

Instructions to authors

Complete this checklist by entering the page numbers from your manuscript where readers will find each of the items listed below.

Your article may not currently address all the items on the checklist. Please modify your text to include the missing information. If you are certain that an item does not apply, please write "n/a" and provide a short explanation.

Upload your completed checklist as an extra file when you submit to a journal.

In your methods section, say that you used the PRISMAreporting guidelines, and cite them as:

Page MJ, McKenzie JE, Bossuyt PM, Boutron I, Hoffmann TC, Mulrow CD, Shamseer L, Tetzlaff JM, Akl EA, Brennan SE, Chou R, Glanville J, Grimshaw JM, Hróbjartsson A, Lalu MM, Li T, Loder EW, Mayo-Wilson E, McDonald S, McGuinness LA, Stewart LA, Thomas J, Tricco AC, Welch VA, Whiting P, Moher D. The PRISMA 2020 statement: An updated guideline for reporting systematic reviews

		Reporting Item	Page Number
**Title**			
Title	#1	Identify the report as a systematic review	1
**Abstract**			
Abstract	#2	Report an abstract addressing each item in the PRISMA 2020 for Abstracts checklist	3
**Introduction**			
Background/rationale	#3	Describe the rationale for the review in the context of existing knowledge	5
Objectives	#4	Provide an explicit statement of the objective(s) or question(s) the review addresses	7
**Methods**			

For peer review only

Eligibility criteria	#5	Specify the inclusion and exclusion criteria for the review and how studies were grouped for the syntheses	7
Information sources	#6	Specify all databases, registers, websites, organisations, reference lists, and other sources searched or consulted to identify studies. Specify the date when each source was last searched or consulted	8
Search strategy	#7	Present the full search strategies for all databases, registers, and websites, including any filters and limits used	8
Selection process	#8	Specify the methods used to decide whether a study met the inclusion criteria of the review, including how many reviewers screened each record and each report retrieved, whether they worked independently, and, if applicable, details of automation tools used in the process	8
Data collection process	#9	Specify the methods used to collect data from reports, including how many reviewers collected data from each report, whether they worked independently, any processes for obtaining or confirming data from study investigators, and, if applicable, details of automation tools used in the process	8
Data items	#10a	List and define all outcomes for which data were sought. Specify whether all results that were compatible with each outcome domain in each study were sought (for example, for all measures, time points, analyses), and, if not, the methods used to decide which results to collect	8
Study risk of bias assessment	#11	Specify the methods used to assess risk of bias in the included studies, including details of the tool(s) used, how many reviewers assessed each study and whether they worked independently, and, if applicable, details of automation tools used in the process	8
Effect measures	#12	Specify for each outcome the effect measure(s) (such as risk ratio, mean difference) used in the synthesis or presentation of results	N/A .As a scoping review, assessment of study heterogeneity,

For peer review only

			and meta-analysis of study results were not performed. Study findings on CYP primary care service use and any associations with patient or caregiver characteristics, were narratively synthesised.
Synthesis methods	#13a	Describe the processes used to decide which studies were eligible for each synthesis (such as tabulating the study intervention characteristics and comparing against the planned groups for each synthesis (item #5))	N/A .As a scoping review, assessment of study heterogeneity, and meta-analysis of study results were not performed. Study findings on CYP primary care service use and any associations with patient or caregiver characteristics, were narratively synthesised.
Synthesis methods	#13b	Describe any methods required to prepare the data for presentation or synthesis, such as handling of missing summary statistics or data conversions	N/A .As a scoping review, assessment of study heterogeneity, and meta-analysis of study results were not performed. Study findings on CYP primary care service use and any associations with patient or caregiver characteristics, were narratively synthesised.

For peer review only

Synthesis methods	#13c	Describe any methods used to tabulate or visually display results of individual studies and syntheses	N/A .As a scoping review, assessment of study heterogeneity, and meta-analysis of study results were not performed. Study findings on CYP primary care service use and any associations with patient or caregiver characteristics, were narratively synthesised.
Synthesis methods	#13d	Describe any methods used to synthesise results and provide a rationale for the choice(s). If meta-analysis was performed, describe the model(s), method(s) to identify the presence and extent of statistical heterogeneity, and software package(s) used	N/A .As a scoping review, assessment of study heterogeneity, and meta-analysis of study results were not performed. Study findings on CYP primary care service use and any associations with patient or caregiver characteristics, were narratively synthesised.
Synthesis methods	#13e	Describe any methods used to explore possible causes of heterogeneity among study results (such as subgroup analysis, meta-regression)	N/A .As a scoping review, assessment of study heterogeneity, and meta-analysis of study results were not performed. Study findings on CYP primary care service use and any associations with

For peer review only

			patient or caregiver characteristics, were narratively synthesised.
Synthesis methods	#13f	Describe any sensitivity analyses conducted to assess robustness of the synthesised results	N/A .As a scoping review, assessment of study heterogeneity, and meta-analysis of study results were not performed. Study findings on CYP primary care service use and any associations with patient or caregiver characteristics, were narratively synthesised.
Reporting bias assessment	#14	Describe any methods used to assess risk of bias due to missing results in a synthesis (arising from reporting biases)	N/A .As a scoping review, assessment of study heterogeneity, and meta-analysis of study results were not performed. Study findings on CYP primary care service use and any associations with patient or caregiver characteristics, were narratively synthesised.
Certainty assessment	#15	Describe any methods used to assess certainty (or confidence) in the body of evidence for an outcome	N/A .As a scoping review, assessment of study heterogeneity, and meta-analysis of study results were not performed. Study

For peer review only

			findings on CYP primary care service use and any associations with patient or caregiver characteristics, were narratively synthesised.
Data items	#10b	List and define all other variables for which data were sought (such as participant and intervention characteristics, funding sources). Describe any assumptions made about any missing or unclear information	8
**Results**			
Study selection	#16a	Describe the results of the search and selection process, from the number of records identified in the search to the number of studies included in the review, ideally using a flow diagram (http://www.prisma-statement.org/PRISMAStatement/FlowDiagram)	9
Study selection	#16b	Cite studies that might appear to meet the inclusion criteria, but which were excluded, and explain why they were excluded	9
Study characteristics	#17	Cite each included study and present its characteristics	9
Risk of bias in studies	#18	Present assessments of risk of bias for each included study	10
Results of individual studies	#19	For all outcomes, present for each study (a) summary statistics for each group (where appropriate) and (b) an effect estimate and its precision (such as confidence/credible interval), ideally using structured tables or plots	11
Results of syntheses	#20a	For each synthesis, briefly summarise the characteristics and risk of bias among contributing studies	N/A .As a scoping review, assessment of study heterogeneity, and meta-analysis of study results were not

For peer review only

			performed. Study findings on CYP primary care service use and any associations with patient or caregiver characteristics, were narratively synthesised.
Results of syntheses	#20b	Present results of all statistical syntheses conducted. If meta-analysis was done, present for each the summary estimate and its precision (such as confidence/credible interval) and measures of statistical heterogeneity. If comparing groups, describe the direction of the effect	N/A .As a scoping review, assessment of study heterogeneity, and meta-analysis of study results were not performed. Study findings on CYP primary care service use and any associations with patient or caregiver characteristics, were narratively synthesised.
Results of syntheses	#20c	Present results of all investigations of possible causes of heterogeneity among study results	N/A .As a scoping review, assessment of study heterogeneity, and meta-analysis of study results were not performed. Study findings on CYP primary care service use and any associations with patient or caregiver characteristics, were narratively synthesised.
Results of syntheses	#20d	Present results of all sensitivity analyses conducted to	N/A .As a scoping

For peer review only

		assess the robustness of the synthesised results	review, assessment of study heterogeneity, and meta-analysis of study results were not performed. Study findings on CYP primary care service use and any associations with patient or caregiver characteristics, were narratively synthesised.
Risk of reporting biases in syntheses	#21	Present assessments of risk of bias due to missing results (arising from reporting biases) for each synthesis assessed	N/A .As a scoping review, assessment of study heterogeneity, and meta-analysis of study results were not performed. Study findings on CYP primary care service use and any associations with patient or caregiver characteristics, were narratively synthesised.
Certainty of evidence	#22	Present assessments of certainty (or confidence) in the body of evidence for each outcome assessed	N/A .As a scoping review, assessment of study heterogeneity, and meta-analysis of study results were not performed. Study findings on CYP primary care service use and any associations with patient or caregiver characteristics, were

For peer review only

			narratively synthesised.
**Discussion**			
Results in context	#23a	Provide a general interpretation of the results in the context of other evidence	15
Limitations of included studies	#23b	Discuss any limitations of the evidence included in the review	17
Limitations of the review methods	#23c	Discuss any limitations of the review processes used	17
Implications	#23d	Discuss implications of the results for practice, policy, and future research	18
**Other information**			
Registration and protocol	#24a	Provide registration information for the review, including register name and registration number, or state that the review was not registered	7
Registration and protocol	#24b	Indicate where the review protocol can be accessed, or state that a protocol was not prepared	7
Registration and protocol	#24c	Describe and explain any amendments to information provided at registration or in the protocol	N/A. No amendments were made to the registered protocol.
Support	#25	Describe sources of financial or non-financial support for the review, and the role of the funders or sponsors in the review	27
Competing interests	#26	Declare any competing interests of review authors	27
Availability of data, code, and other materials	#27	Report which of the following are publicly available and where they can be found: template data collection forms; data extracted from included studies; data used for all analyses; analytic code; any other materials used in the review	8,10

Notes:

For peer review only

- 12: N/A .As a scoping review, assessment of study heterogeneity, and meta-analysis of study results were not performed. Study findings on CYP primary care service use and any associations with patient or caregiver characteristics, were narratively synthesised.
- 13a: N/A .As a scoping review, assessment of study heterogeneity, and meta-analysis of study results were not performed. Study findings on CYP primary care service use and any associations with patient or caregiver characteristics, were narratively synthesised.
- 13b: N/A .As a scoping review, assessment of study heterogeneity, and meta-analysis of study results were not performed. Study findings on CYP primary care service use and any associations with patient or caregiver characteristics, were narratively synthesised.
- 13c: N/A .As a scoping review, assessment of study heterogeneity, and meta-analysis of study results were not performed. Study findings on CYP primary care service use and any associations with patient or caregiver characteristics, were narratively synthesised.
- 13d: N/A .As a scoping review, assessment of study heterogeneity, and meta-analysis of study results were not performed. Study findings on CYP primary care service use and any associations with patient or caregiver characteristics, were narratively synthesised.
- 13e: N/A .As a scoping review, assessment of study heterogeneity, and meta-analysis of study results were not performed. Study findings on CYP primary care service use and any associations with patient or caregiver characteristics, were narratively synthesised.
- 13f: N/A .As a scoping review, assessment of study heterogeneity, and meta-analysis of study results were not performed. Study findings on CYP primary care service use and any associations with patient or caregiver characteristics, were narratively synthesised.
- 14: N/A .As a scoping review, assessment of study heterogeneity, and meta-analysis of study results were not performed. Study findings on CYP primary care service use and any associations with patient or caregiver characteristics, were narratively synthesised.
- 15: N/A .As a scoping review, assessment of study heterogeneity, and meta-analysis of study results were not performed. Study findings on CYP primary care service use and any associations with patient or caregiver characteristics, were narratively synthesised.
- 20a: N/A .As a scoping review, assessment of study heterogeneity, and meta-analysis of study results were not performed. Study findings on CYP primary care service use and any associations with patient or caregiver characteristics, were narratively synthesised.
- 20b: N/A .As a scoping review, assessment of study heterogeneity, and meta-analysis of study results were not performed. Study findings on CYP primary care service use and any associations with patient or caregiver characteristics, were narratively synthesised.

For peer review only

- 20c: N/A .As a scoping review, assessment of study heterogeneity, and meta-analysis of study results were not performed. Study findings on CYP primary care service use and any associations with patient or caregiver characteristics, were narratively synthesised.
- 20d: N/A .As a scoping review, assessment of study heterogeneity, and meta-analysis of study results were not performed. Study findings on CYP primary care service use and any associations with patient or caregiver characteristics, were narratively synthesised.
- 21: N/A .As a scoping review, assessment of study heterogeneity, and meta-analysis of study results were not performed. Study findings on CYP primary care service use and any associations with patient or caregiver characteristics, were narratively synthesised.
- 22: N/A .As a scoping review, assessment of study heterogeneity, and meta-analysis of study results were not performed. Study findings on CYP primary care service use and any associations with patient or caregiver characteristics, were narratively synthesised.
- 24c: N/A. No amendments were made to the registered protocol. The PRISMA checklist is distributed under the terms of the Creative Commons Attribution License CC-BY. This checklist was completed on 03. August 2023 using https://www.goodreports.org/, a tool made by the EQUATOR Network in collaboration with Penelope.ai

For peer review only

Patient and caregiver characteristics associated with differential use of primary care for children and young people in the UK: a scoping review

Journal:	*BMJ Open*
Manuscript ID	bmjopen-2023-078505.R1
Article Type:	Original research
Date Submitted by the Author:	19-Mar-2024
Complete List of Authors:	Herbert, Kevin; Cambridge University, Cambridge Research Methods Hub, Department of Public Health and Primary Care,<br>Herlitz, Lauren; UCL GOS Institute of Child Health, Population, Policy and Practice Department,<br>Woodman, Jenny; UCL Social Research Institute, Institute of Education,<br>Powell, Claire; UCL GOS Institute of Child Health, Population, Policy and Practice Department,<br>Morris, Stephen; University of Cambridge, Cambridge Research Methods Hub, Department of Public Health and Primary Care,
**Primary Subject Heading**:	General practice / Family practice
Secondary Subject Heading:	Health services research, Dentistry and oral medicine
Keywords:	Primary Care < Primary Health Care, Health Equity, Adolescent, Health Services Accessibility

SCHOLARONE™ Manuscripts

I, the Submitting Author has the right to grant and does grant on behalf of all authors of the Work (as defined in the below author licence), an exclusive licence and/or a non-exclusive licence for contributions from authors who are: i) UK Crown employees; ii) where BMJ has agreed a CC-BY licence shall apply, and/or iii) in accordance with the terms applicable for US Federal Government officers or employees acting as part of their official duties; on a worldwide, perpetual, irrevocable, royalty-free basis to BMJ Publishing Group Ltd ("BMJ") its licensees and where the relevant Journal is co-owned by BMJ to the co-owners of the Journal, to publish the Work in this journal and any other BMJ products and to exploit all rights, as set out in our licence.

The Submitting Author accepts and understands that any supply made under these terms is made by BMJ to the Submitting Author unless you are acting as an employee on behalf of your employer or a postgraduate student of an affiliated institution which is paying any applicable article publishing charge ("APC") for Open Access articles. Where the Submitting Author wishes to make the Work available on an Open Access basis (and intends to pay the relevant APC), the terms of reuse of such Open Access shall be governed by a Creative Commons licence – details of these licences and which Creative Commons licence will apply to this Work are set out in our licence referred to above.

Other than as permitted in any relevant BMJ Author's Self Archiving Policies, I confirm this Work has not been accepted for publication elsewhere, is not being considered for publication elsewhere and does not duplicate material already published. I confirm all authors consent to publication of this Work and authorise the granting of this licence.

For peer review only

Patient and caregiver characteristics associated with differential use of primary care for children and young people in the UK: a scoping review

For peer review only

For peer review only - http://bmjopen.bmj.com/site/about/guidelines.xhtml

Patient and caregiver characteristics associated with differential use of primary care for children and young people in the UK: a scoping review

Authors and Affiliations

Kevin Herbert, ORCID 0009-0008-4354-7811* Cambridge Research Methods Hub, Department of Public Health and Primary Care, East Forvie Building, Forvie Site, Robinson Way, Cambridge CB2 0SR. Email: kch28@medschl.cam.ac.uk

Lauren Herlitz, ORCID 0000-0003-2497-9041. UCL GOS Institute of Child Health, Population, Policy and Practice Department, 30 Guilford Street, London. WC1N 1EH, UK

Jenny Woodman, ORCID 0000-0002-9403-4177. UCL Social Research Institute, Institute of Education, 27 Woburn Sq, London. WC1H 0AA, UK

Claire Powell, ORCID 0000-0002-6581-0165. UCL GOS Institute of Child Health, Population, Policy and Practice Department, 30 Guilford Street, London. WC1N 1EH, UK

Stephen Morris, ORCID 0000-0002-5828-3563. Cambridge Research Methods Hub, Department of Public Health and Primary Care, East Forvie Building, Forvie Site, Robinson Way , Cambridge CB2 0SR.

*Corresponding author

For peer review only

Abstract (max 300 words)

Objective: To systematically map evidence to answer the research question: *What is the relationship between the characteristics of children and young people (CYP) or their caregivers and primary care service use in the UK, taking into account underlying healthcare needs?*

Design: Scoping review.

Setting: Primary care.

Eligibility criteria: English-language quantitative or mixed-methods studies published between 2012 and 2022.

Data sources: Medline, Embase, Scopus and Web of Science Social Sciences Citation Index, and grey literature.

Results: Twenty-two eligible studies were identified, covering general practice (n=14), dental health (n=4), child mental health services (n=3), and immunisation (n=1). Only eight studies (36%) controlled for variables associated with healthcare need (e.g., age, birthweight, long-term conditions). In these, evidence of horizontal inequity in primary care use was reported for CYP living in deprived areas in England, with and without complex needs. Horizontal inequity was also identified in primary care mental health referrals for CYP in England identifying as mixed-race, Asian, or black ethnicity, compared to their White British peers. No evidence of horizontal inequity was observed, however, in primary care use for CYP in England exposed to parental depression, or for CYP children from low-income households in Scotland. Increasing CYP's age was associated with decreasing primary care use across included studies. No studies were found regarding CYP from Gypsy or Traveller communities, children in care, those with disabilities or special educational needs.

Conclusions: There is evidence that socio-economic factors impact upon CYP's primary care use, in particular age, ethnicity, and deprivation. However, better quality evidence is required to evaluate

For peer review only

horizontal inequity in use, and address knowledge gaps regarding primary care use for vulnerable CYP populations and the impact of policy and practice related 'supply side' of primary care.

Key words

Primary care; adolescents; children; access to healthcare; health equity; horizontal inequity; unmet need

Strengths and limitations (up to 5 bullet points)

- Through the detailed data extraction of included studies, this review comprehensively documents the study populations, healthcare settings and CYP/caregiver characteristics considered to explore the use of primary care by CYP in the UK.
- By restricting eligible publications to the last decade, the findings reported are relevant to current healthcare systems and social context.
- Through the use of systematic methodology – including publication quality appraisal – this review was designed and conducted to ensure robustness and reproducibility.
- As a scoping review, this review and its findings should not however, be treated as exhaustive.

For peer review only - http://bmjopen.bmj.com/site/about/guidelines.xhtml

Introduction

In recent years, health inequality (avoidable differences in health outcomes between groups) has been growing amongst the UK population (1,2). For children and young people (CYP) in England, those from Pakistani, Black African and Black Caribbean ethnic groups experience higher rates of infant mortality than other ethnicities (1). Vulnerable CYP (e.g., those with a learning disability or autism) have worse health and wellbeing outcomes, service experiences, poor outcomes associated with chronic conditions, and greater premature mortality risk (3–6). In recognition, the NHS England long term plan highlights the role of primary care in reducing health inequalities (7).

Horizontal inequity is defined as the unequal treatment for people with equal need (8). Despite evidence of horizontal inequity across the UK adult population (9,10), inequities amongst CYP is less well understood, particularly in primary care. This evidence gap is of particular concern given its potential to inhibit the effective development of policies to ensure that health care provision effectively meets CYP's level of healthcare need.

Analysis of routinely collected national data, indicates that infants and preschool children have the highest general practice visit rates of any age group (11,12), whilst in the year 2021-22, nearly half of children aged 0 to 17 years in England receive NHS dental care annually (13). Beyond dental service access by patient age however, there are no government-provided national CYP statistics on the use of primary care use, or how their use varies for different sub-populations relative to healthcare need.

In England, policies have been introduced over the last 30 years to specifically tackle deprivation-based inequity in general practice (14). Despite some evidence of improvements, their effectiveness may have been impaired by inadequate compensation for additional deprivation-related healthcare needs via the core general practice funding formula (14).

For peer review only - http://bmjopen.bmj.com/site/about/guidelines.xhtml

To effectively address equity within primary care and inform policy-making relating to the supply of care (e.g., service quality, quantity, and distribution) the best available evidence is needed for both vulnerable and marginalised CYP groups, and the CYP population as a whole. In this article, we examine existing evidence on CYP primary care use and ask: what is the relationship between the characteristics of CYP or their caregivers and primary care service use in the UK, taking into account underlying healthcare needs?

Method

The design of the review was informed by the Preferred Reporting Items for Systematic Reviews and Meta-Analysis extension for scoping reviews (PRISMA-ScR) guidance for rapid evidence reviews, developed by Tricco *et al*. (2018) (15). The protocol was registered in the Open Science Framework (Centre for Open Science, https://osf.io/mfc3z) and followed the PRISMA-ScR guide for the review design and reporting of methods and findings, and associated checklist (16). Due to the rapid nature of the review (10 weeks), the questions and search strategy were targeted to identify relevant articles that could be analysed within the review timeframe.

Inclusion/exclusion criteria

A study was included if it:

- Focused on the characteristics of CYP (aged 0 to 25 years), or families of children (aged 0 to 18 years);
- Was conducted on data for UK primary health care settings (general practice, community pharmacy, NHS provided dentistry);
- Outcomes were reported in terms of primary care use measures (e.g., primary care contacts, NHS dentist registration);
- Used quantitative or mixed-methods empirical methods;
- Was published between January 1st 2012 and 5th July 2022;

For peer review only - http://bmjopen.bmj.com/site/about/guidelines.xhtml

Studies were excluded if they:

- Focused on school-based health services (e.g., school nursing);

Search strategy

We used free-text and controlled terms (Appendix A) to search four electronic databases Medline, Embase, Scopus and Web of Science (Social Sciences Citation Index). To identify publications not indexed in these main databases, an additional 100 publications identified from a grey literature search using Google Scholar, were screened (17,18).

Document selection

The search results were imported into Rayyan software (https://www.rayyan.ai/) for de-duplication, before being divided equally between two reviewers and screened independently by title and abstract. 10% of excluded articles for each reviewer were cross-checked by the other, to confirm agreement. As all excluded articles were agreed between reviewers at this stage, screening proceeded to a full text review by a single reviewer, with any articles for query referred to a second reviewer.

Data extraction and quality assessment

From included studies, two reviewers extracted: study sample/population; health care setting; area of health care; study design; methodology; variables controlling for healthcare need (where applicable); primary care contacts. We also extracted data for outpatient attendance, specialist referrals, and hospital-based service use (e.g., emergency presentations; hospital admissions) to explore potential associations between CYP primary care use and other health services. Study quality was assessed using the Mixed Methods Appraisal Tool (MMAT), (19,20). No study was excluded based on quality, but we report study quality.

For peer review only

For peer review only - http://bmjopen.bmj.com/site/about/guidelines.xhtml

Data synthesis

As a scoping review, assessment of study heterogeneity, and meta-analysis of study results were not performed. Study findings on CYP primary care service use and any associations with patient or caregiver characteristics, were narratively synthesised.

Patient and Public Involvement

As a scoping review of published literature, no patients were involved in the design, conduct or analysis of this research.

Results

We found 2,301 unique title/abstracts, of which 22 publications (reporting 21 studies) met the inclusion criteria (Figure 1)

Study characteristics

Study origin, health care setting and area of health

Most studies related to English primary care services (n=17); the rest studied primary care in Northern Ireland (n=2), Wales (n=1), Ireland and Scotland (n=1), and the UK (n=1) (see Appendix B). Most focused upon utilisation of general practice services (n=14), with the remaining, dental services or oral health (n=4), child mental health services (n=3), and the uptake of immunisations (n=1).

Study designs and samples

Most of the eligible publications reported on cohort studies (n=17), with the remaining cross-sectional studies (n=5). The most common age ranges of CYP studied were 0 to 15 years (n=3), 0 to 18 years (n=3), 0 to 1 year (n=2), and 0 to 14 years (n=2) (see Figure 2 and **Error! Reference source not found.**).

For peer review only - http://bmjopen.bmj.com/site/about/guidelines.xhtml

For peer review only

Characteristics studied

Eligible studies predominately investigated deprivation or social economic differences (n=16), sex or gender (n=13), ethnic group (n=11) or age (n=8) for potential associations with levels of primary care service use. Five of the included studies compared primary care use for CYP with a defined health condition: congenital abnormalities; CYP mental health; Down's syndrome; eczema (**Error! Reference source not found.**).

Fifteen of the included studies contained one or more healthcare need variables in their analysis of CYP primary care use (**Error! Reference source not found.**). Eight studies controlled for healthcare need in a general population sample, with CYP age (n=8), gestational age (n=3), and birthweight (n=2) the most often controlled for in regression analyses. Seven studies compared use between subsets of the primary CYP sample, the most commonly investigated subsets were CYP age (n=7), CYP with comorbidities (n=2), or CYP with defined conditions (n=2). None of the included studies accounted for variation in supply (e.g., CYP distance from practices, general practitioner [GP] appointment availability, GP to patient ratio) in the analysis of CYP use of primary care.

Outcomes

Table 1 summarises health care utilisation outcomes reported by included studies. The services most investigated were primary care attendance and/or GP consultations (n=13), outpatient attendances (n=7) and specialist referrals (n=5). Use of non-primary care services was recorded in nine of the eligible studies (see Table 1), to study their use in addition to primary care or to explore possible relationships between primary care presentations and the need for hospital-based care.

The relationship between CYP characteristics and use of publicly funded dental health services was investigated in four of the included studies. Outcomes reported for these studies focused upon the registration or rates of attendance with NHS (England) or General Dental Service (Northern Ireland)

For peer review only - http://bmjopen.bmj.com/site/about/guidelines.xhtml

dental healthcare services, dental or oral health by examination, or publicly funded fee for service reimbursement for dental care provided (see Table 1).

Study quality

All studies bar one met four or five of the MMAT quality criteria (see **Error! Reference source not found.**). Fifteen of the twenty-two eligible publications included a statistical analysis and/or a study sample which accounted for healthcare need. Of these, eight high quality publications controlled for healthcare need in their analysis. The remaining seven publications were assigned high quality as their study designs were appropriate for their respective research questions, but we noted that indicators of healthcare need as criteria were only used for deriving study samples (for full quality criteria, see Appendix C).

CYP or caregiver characteristics affecting primary care use

The next sections report the CYP or caregiver factors associated with UK primary care use. Although some characteristics were examined in multiple studies (for example, CYP age), or with large samples (for example, parental mental health), overall, the literature was fragmented with one or two studies reporting findings for some characteristics. Findings related to general practice are reported first in each section, followed by use of dental services. We have highlighted findings from high quality studies which have controlled for healthcare need variables.

Sex or gender of CYP

Some evidence was apparent for gender-based differences in the use of primary care, and the routes by which CYP are referred to specialist services. Two high quality studies that controlled respectively for age, and age and type of mental health problem, found that males were more likely to be referred to child and adolescent mental health services (CAMHS) via education services than via primary care, compared to their female counterparts (21,22). Female CYP with a parental history of

For peer review only - http://bmjopen.bmj.com/site/about/guidelines.xhtml

recurrent depression, were more likely to use primary care for the treatment of their own anxiety disorder, than their male counterparts (23). In contrast, in a study that characterised different patterns of CYP healthcare use in Northwest London, males were more likely to appear in the high service use cluster compared to females, after adjusting for age, ethnicity, deprivation and long-term conditions (24).

Where dental services were studied, females were found to be more likely to use orthodontic services than males (25). Access to dental services also became differentiated with age with females being more likely to be registered with an NHS dentist than males by the age of 15-16 years, from no difference at 11-12 years of age (26).

Age of CYP

Consistent across included studies, increasing age of the child was associated with a fall in the use of primary care services, with older teenagers less likely to use primary care (12,24,26–28).

Deprivation or socio-economic classification

CYP from areas of higher deprivation in England were reported to have had lower levels of GP consultations, considering both CYP in the general population or CYP with congenital abnormalities (29,30). This was despite the overall need for health care being higher, reflected in a shift from the routine use of scheduled care services (e.g., GP consultations and outpatient attendance) to those that deal with unscheduled presentations (e.g., ED attendance), or hospital admissions. Exposure to deprivation was also observed to affect the uptake and timeliness of child vaccinations, with lower rates of uptake and later vaccinations for children from areas of highest deprivation (31). Of note, family GP practices with highest reported accessibility according to the GP Patient Survey were more likely to be located in more affluent areas, less likely to be in urban areas, and also had a lower proportion of their registered patient list being children (32).

For peer review only - http://bmjopen.bmj.com/site/about/guidelines.xhtml

In contrast to the above, the study of different patterns of health care use in Northwest London found higher levels of deprivation in clusters of CYP that had higher health service utilisation, including primary care (24). This finding indicates that a minority of CYP from the most deprived areas may be frequent attenders of primary care, but in general, more CYP from deprived areas would be expected to have higher rates of primary care service use due to higher levels of healthcare need. It should also be considered that the Beaney *et al*. (2021) study may not be nationally representative of the UK CYP population, due to the study population being drawn from an area with higher ethnic diversity and levels of deprivation, relative to national averages (24).

One high quality study that accounted for CYP age, birthweight, and parental assessment of CYP health among other factors, explored family income as a possible factor in inequality of GP service utilisation for samples of children from Scotland and the Republic of Ireland (33). We report only on the UK-related findings. The authors identified little or no overall income-related inequity in GP care for Scottish children in the overall analysis. The probability of GP visits was higher in younger children (2-year-olds) from less deprived backgrounds, although this was almost totally negated after adjustment for health and non-need determinants.

In dental care, children in the lowest socio-economic status group were more likely to require reparative dental treatments, and dental treatment overall, but were less likely to have consumed orthodontic services (26,34).

For children with congenital abnormalities, higher maternal education was associated with lower levels of primary care consultations and hospital service use - potentially a consequence of less educated parents requiring additional help from medical professionals to manage CYP complex needs (30). In contrast, lower educational attainment of the household reference person was associated with CYP having less time registered with an NHS dentist (26), and where the reference person had no qualifications, more than twice was spent on endodontics for the CYP than where a degree or greater was obtained (25).

For peer review only - http://bmjopen.bmj.com/site/about/guidelines.xhtml

Ethnic background

Associations were identified between ethnic background and primary care use, which were moderated by intersectionality. Three high quality studies, including one that controlled for age, birthweight and gestational age (Hasan & West, 2021), reported that children from Black, Pakistani, Asian or Asian British ethnicity were observed to have GP consultation or prescription rates at higher levels than the majority population, while outpatient attendance and other services were relatively underutilised (24,29,35).

Babies born at term with low birth weights are recognised as having increased morbidity and mortality though infancy to adulthood (36). Some ethnic minorities (Indian, Bangladeshi, Pakistani, Black Caribbean, or Other) are observed to be overrepresented in terms of babies delivered at low birthweights (1). However, one high quality study that accounted for CYP gestational age, found that children of Pakistani ethnicity had greater primary care use than White British children irrespective of birthweight (37).

Two high quality studies that controlled respectively for age and type of mental health problem, found that children of Black, Asian, other (non-White), White other, or of mixed race, were more likely to be referred to child mental health services through routes other than primary care (e.g., education services, social services, youth justice), relative to their White British counterparts (21,22). Children of Asian, Black, White other, or other ethnicities were also less likely to have their case closed through non-attendance – a finding thought to be related to the higher rate of compulsory child mental health referrals (21,22).

Dental services reflected poorer outcomes in children of ethnic minorities, with those of White European, Bangladeshi or Pakistani ethnicity more likely to have poor dental health (tooth decay, teeth missing due to caries, fillings score, or greater numbers of untreated carious teeth), relative to children identifying as White British (38).

For peer review only - http://bmjopen.bmj.com/site/about/guidelines.xhtml

CYP with existing conditions

Differences in primary care service use were observed for children with existing conditions. One high quality study that controlled for multimorbidity found that CYP of Pakistani or 'Other' ethnic origin with congenital abnormalities were found to have higher primary care use compared to their White British peers (30). A greater chance of eczema treatment escalation was also seen for CYP of Asian, Black African/Caribbean, Mixed or Other ethnicities, relative to those of White ethnicity (39). It is possible that these variations may be reflective of: condition-specific differences for different population subgroups; the performance of treatment pathways; GP-patient relationships; parental attitudes; or their presentation thresholds for primary care attendance. For children with Down's syndrome (DS), higher rates of primary care use (GP consultations, prescriptions) due to respiratory tract infections (RTIs) were observed, relative to a matched control group without DS (40). The authors noted that this may at least in part be due to increased RTI susceptibility and severity in children with DS, arising from vulnerabilities caused by differences in immunology, and airway morphology and function.

Parental MH

Two high quality studies, one of which accounted for CYP's age and presence of chronic conditions, found that CYP exposure to maternal mental illness was found to be more prevalent with increased deprivation, and associated with increased CYP primary health care use (27,41). Maternal mental illness exposed CYP had higher rates of primary care service contacts, outpatient attendance and specialist referrals. They also received more drug prescriptions for mental and behavioural disorders, physical, acute, or chronic diseases. Across all CYP who accessed primary care services, use was greater for those with mothers who had poor mental health than those without, which may indicate differences in health seeking behaviour or a higher level of healthcare need (27,41).

For peer review only - http://bmjopen.bmj.com/site/about/guidelines.xhtml

In contrast, Potter *et al*. (2012) did not find any effect of having parents with current mental health conditions on the use of primary care by CYP (23). It should be noted however, that this study was based upon a relatively small sample size (n=333), compared to those used in Hope *et al*. (2021) and Dreyer *et al*. (2018) (n=489,255 and n=25,252 respectively) (27,41).

Migrant status

Eligible studies for primary care access and use by children with migrant backgrounds was limited. One high quality study that controlled for CYP age, reported that children of Indian ethnicity with a migrant background, were less likely to self-refer for mental health issues, although no differences in overall use of mental health services was observed (42,43).

Refugee status

Only one study (of lower quality) was identified on primary care use by a sample of children with refugee status of varied ethnicities (Afghanistan, Ethiopia, Gambia, Iran, Iraq, East Africa, Kenya, Nigeria, Pakistan, Somalia, Sudan, Tunisia, Vietnam, Zambia, Zimbabwe). No difference was found for medicines consumption relative to the control group studied (44), although evidence did indicate that over the counter, rather than prescribed medicines were more likely to be the primary source for refugee children.

Discussion

While the review identified multiple studies of CYP primary care use, relatively few took into account indicators of CYP's underlying healthcare needs. Studies consistently found lower rates of CYP primary care use with increasing CYP age. There was evidence that CYP living in deprived areas in England used primary care less than their counterparts living in wealthier areas, a finding identified by both studies that accounted for indicators of underlying healthcare needs and those that compared subsets of CYP. There was some evidence to suggest that CYP in deprived areas were

For peer review only - http://bmjopen.bmj.com/site/about/guidelines.xhtml

For peer review only

more likely to use acute care services. Recent research has identified key principles to reduce the likelihood of health inequalities resulting from general practice, including accounting for differences within patient groups (45).

Included studies also suggested horizontal inequity in referrals to mental health services. Primary care services were less likely to refer male or non-White British CYP to CAMHS, potentially as a result of differences in help-seeking preferences, patient engagement, or 'GP gatekeeping' (46), highlighting an issue for which service and/or patient-focused solutions are being sought (47–49). Where NHS dental services were studied, a need for greater oral care education and outreach was indicated by the poor dental health and service access for CYP of non-White British ethnicity, and those from low socio-economic status and poorly educated households (26,34,38).

Two thirds of the studies (n=14) did not control for underlying healthcare need in their analyses, and as a consequence it is difficult to understand whether differences in primary health care use reflect service inequalities or variation that is appropriately based on differences in need. Where studies did account for health indicators, age, gestational age and birthweight were the most commonly controlled for variables. Morbidity or CYP health status were largely absent from analyses. With regards other factors that may influence CYP primary care use, little reference was made to supply-side issues in general practice (e.g., appointment availability, practice location, GP to patient ratio), outside of a single study focused on accessibility via responses to the GP Patient Survey. Whilst the growing pressures on general practice are well reported (50), how these affect the CYP population and specifically how they may drive inequities in service use remains unclear.

Key gaps in the knowledge landscape were also identified. It should be noted that all included studies focused on CYP registered with primary care providers, with no studies found for unregistered patients, or service use in other care settings (e.g., walk-in centres), pointing toward a paucity in data collection in these areas. Little quantitative evidence was found regarding CYP primary care use in refugee or migrant populations, whilst no studies investigated primary care use

For peer review only - http://bmjopen.bmj.com/site/about/guidelines.xhtml

for CYP from Gypsy or Traveller communities, care-experienced CYP, or for CYP with learning disabilities, autism or special educational needs.

This review was strengthened through clearly defined scoping review protocols, thus optimising search, screening and data extraction processes and ensuring robustness and reproducibility. Through the detailed extraction of data from included studies, this review comprehensively documents the study populations, healthcare settings and CYP/caregiver characteristics considered to explore the use of primary care by CYP in the UK. By confining eligible publications to those from within the last decade, this study also focuses on evidence relevant to current social dynamics and clinical practice.

To inform the wider research project on CYP's access and use of primary care, we took a pragmatic approach to the timeframe for the literature review, conducting the review rapidly while retaining elements required for a robust review. It is understood that there is debate regarding the value of Google Scholar in systematic reviewing, (17) however it was felt important to include a search of grey literature, due to the focus of the study – aspects of which (e.g., horizontal inequity) are of particular interest to organisations who produce research reports which would not fall within the scope of the key literature databases. Limiting the main search to key literature databases maximised the coverage of potentially relevant publications in the time available and allowed for detailed data extraction and analysis from the publications identified. Consequently, the findings should not be treated as exhaustive.

Conclusion

Recent evidence suggests that CYP age, deprivation, ethnicity, and gender influence CYP primary care use, including NHS dental services. Studies that have accounted for indicators of healthcare need suggest that CYP living in deprived areas in England use primary care services less than CYP in wealthier areas with equal levels of need and have greater use of acute care. Despite these findings, our assessment is that better quality evidence is required to adequately evaluate horizontal inequity

For peer review only - http://bmjopen.bmj.com/site/about/guidelines.xhtml

in CYP primary care use and address knowledge gaps regarding primary care use for vulnerable CYP populations, as well as the impact of 'supply side' policy and practice for the delivery of high-quality primary care services to all CYP.

For peer review only

For peer review only - http://bmjopen.bmj.com/site/about/guidelines.xhtml

References

1. Public Health England. Local action on health inequalities. Understanding and reducing ethnic inequalities in health. 2018.

2. Marmot M, Allen J, Boyce T, Goldblatt P, Morrison J. Health equity in England: The Marmot review 10 years on. 2020.

3. Glover G, Williams R, Branford D, Avery R, Chauhan U, Hoghton M, et al. Prescribing of psychotropic drugs to people with learning disabilities and/or autism by general practitioners in England. 2015.

4. Crane L, Chester JW, Goddard L, Henry LA, Hill E. Experiences of autism diagnosis: A survey of over 1000 parents in the United Kingdom. Autism. 2015 Mar 25;20(2):153–62.

5. Department of Health and Social Care. The Government response to the Learning Disabilities Mortality Review (LeDeR) Programme Second Annual Report. 2016.

6. Wijlaars LPMM, Gilbert R, Hardelid P. Chronic conditions in children and young people: learning from administrative data. Arch Dis Child. 2016 Oct 1;101(10):881 LP – 885.

7. NHS England. The NHS Long Term Plan. 2019.

8. Pulok MH, van Gool K, Hajizadeh M, Allin S, Hall J. Measuring horizontal inequity in healthcare utilisation: a review of methodological developments and debates. Eur J Heal Econ. 2020;21(2):171–80.

9. Cookson R, Propper C, Asaria M, Raine R. Socio-Economic Inequalities in Health Care in England. Fisc Stud. 2016 Sep 1;37(3–4):371–403.

10. Turner AJ, Francetic I, Watkinson R, Gillibrand S, Sutton M. Socioeconomic inequality in access to timely and appropriate care in emergency departments. J Health Econ. 2022;85:102668.

11. Hobbs FDR, Bankhead C, Mukhtar T, Stevens S, Perera-Salazar R, Holt T, et al. Clinical workload in UK primary care: a retrospective analysis of 100 million consultations in England, 2007–14. Lancet. 2016;387(10035):2323–30.

12. Ruzangi J, Blair M, Cecil E, Greenfield G, Bottle A, Hargreaves D. Trends in healthcare use in children aged less than 15 years: A population-based cohort study in England from 2007 to 2017. BMJ Open. 2020;10(5):e033761.

13. NHS Digital. NHS Dental Statistics for England dashboard [Internet]. 2022 [cited 2023 Mar 29]. Available from: https://digital.nhs.uk/data-and-information/data-tools-and-services/data-services/general-practice-data-hub/dentistry

14. The Health Foundation. Tackling the inverse care law. Analysis of policies to improve general practice in deprived areas since 1990. 2022.

15. Tricco AC, Lillie E, Zarin W, O'Brien KK, Colquhoun H, Levac D, et al. PRISMA extension for scoping reviews (PRISMA-ScR): Checklist and explanation. Ann Intern Med. 2018;169(7):467–73.

16. Page MJ, McKenzie JE, Bossuyt PM, Boutron I, Hoffmann TC, Mulrow CD, et al. The PRISMA 2020 statement: an updated guideline for reporting systematic reviews. BMJ. 2021 Mar

For peer review only - http://bmjopen.bmj.com/site/about/guidelines.xhtml

29;372:n71.

17. Haddaway NR, Collins AM, Coughlin D, Kirk S. The Role of Google Scholar in Evidence Reviews and Its Applicability to Grey Literature Searching. PLoS One. 2015 Sep 17;10(9):e0138237.

18. Halevi G, Moed H, Bar-Ilan J. Suitability of Google Scholar as a source of scientific information and as a source of data for scientific evaluation—Review of the Literature. J Informetr. 2017;11(3):823–34.

19. Pace R, Pluye P, Bartlett G, Macaulay AC, Salsberg J, Jagosh J, et al. Testing the reliability and efficiency of the pilot Mixed Methods Appraisal Tool (MMAT) for systematic mixed studies review. Int J Nurs Stud. 2012;49(1):47–53.

20. Souto RQ, Khanassov V, Hong QN, Bush PL, Vedel I, Pluye P. Systematic mixed studies reviews: Updating results on the reliability and efficiency of the mixed methods appraisal tool. Int J Nurs Stud. 2015;52(1):500–1.

21. Edbrooke-Childs J, Newman R, Fleming I, Deighton J, Wolpert M. The association between ethnicity and care pathway for children with emotional problems in routinely collected child and adolescent mental health services data. Eur Child Adolesc Psychiatry. 2016;25(5):539–46.

22. Edbrooke-Childs J, Patalay P. Ethnic Differences in Referral Routes to Youth Mental Health Services. J Am Acad Child Adolesc Psychiatry. 2019;58(3):368-375.e1.

23. Potter R, Mars B, Eyre O, Legge S, Ford T, Sellers R, et al. Missed opportunities: Mental disorder in children of parents with depression. Br J Gen Pract. 2012;62(600):e487–93.

24. Beaney T, Clarke J, Woodcock T, McCarthy R, Saravanakumar K, Barahona M, et al. Patterns of healthcare utilisation in children and young people: A retrospective cohort study using routinely collected healthcare data in Northwest London. BMJ Open. 2021;11(12).

25. Telford C, Murray L, Donaldson M, O'Neill C. An analysis examining socio-economic variations in the provision of NHS general dental practitioner care under a fee for service contract among adolescents: Northern Ireland Longitudinal Study. Community Dent Oral Epidemiol. 2012;40(1):70–9.

26. Telford C, O'Neill C. Changes in dental health investment across the adolescent years. Br Dent J. 2012;212(9).

27. Hope H, Osam C, Kontopantelis E, Hughes S, Munford L, Ashcroft D, et al. The healthcare resource impact of maternal mental illness on children and adolescents: UK retrospective cohort study. Br J Psychiatry. 2021;219(3):515–22.

28. Manikam L, Cupp M, Schilder A, Hayward A, Littlejohns P, Alexander E. Respiratory tract infection related healthcare utilisation in UK children with down's syndrome. Am J Respir Crit Care Med. 2018;197(MeetingAbstracts).

29. Coughlan C, Ruzangi J, Neale F, Nezafat Maldonado B, Blair M, Bottle A, et al. Social and ethnic group differences in healthcare use by children aged 0-14 years: A population-based cohort study in England from 2007 to 2017. Arch Dis Child. 2022;107(1):32–9.

30. Bishop C, Small N, Parslow R. Healthcare use for children with complex needs: Using routine health data linked to a multiethnic, ongoing birth cohort. BMJ Open. 2018;8(3):e018419.

31. Perry M, McGowan A, Roberts R, Cottrell S. Timeliness and equity of infant pertussis

For peer review only - http://bmjopen.bmj.com/site/about/guidelines.xhtml

vaccination in wales: Analysis of the three dose primary course. Vaccine. 2020;38(6):1402–7.

32. Cecil E, Bottle A, Cowling TE, Majeed A, Wolfe I, Saxena S. Primary care access, emergency department visits, and unplanned short hospitalizations in the UK. Pediatrics. 2016;137(2).

33. Layte R, Nolan A. Income-related inequity in the use of GP services by children: a comparison of Ireland and Scotland. Eur J Health Econ. 2015;16(5):489–506.

34. Salomon-Ibarra C, Ravaghi V, Hill K, Jones C, Landes D, Morris A. Low rates of dental attendance by the age of one and inequality between local government administrative areas in England. Community Dent Health. 2019;36(1):22–6.

35. Hasan T, West J. Body mass index and use and costs of primary care services among white british and pakistani children: Findings from the born in bradford cohort study. Arch Dis Child. 2021;106(SUPPL 1):A393–4.

36. Watkins WJ, Kotecha SJ, Kotecha S. All-Cause Mortality of Low Birthweight Infants in Infancy, Childhood, and Adolescence: Population Study of England and Wales. PLOS Med. 2016 May 10;13(5):e1002018.

37. West J, Kelly B, Collings P, Santorelli G, Mason D. Is small size at birth associated with early childhood morbidity in white British and Pakistani origin UK children aged 0-3? Findings from the born in Bradford cohort study. BMC Pediatr. 2018;18(1):22.

38. Marcenes W, Muirhead VE, Murray S, Redshaw P, Bennett U, Wright D. Ethnic disparities in the oral health of three- to four-year-old children in East London. Br Dent J. 2013;215(2).

39. de Lusignan S, Alexander H, Broderick C, Dennis J, McGovern A, Feeney C, et al. Patterns and trends in eczema management in UK primary care (2009-2018): A population-based cohort study. Clin Exp Allergy. 2021;51(3):483–94.

40. Manikam L, Schilder AGM, Lakhanpaul M, Littlejohns P, Alexander EC, Hayward A. Respiratory tract infection-related healthcare utilisation in children with Down's syndrome. Infection. 2020;48(3):403–10.

41. Dreyer K, Williamson R, Hargreaves D, Rosen R. Associations between parental mental health and other family factors and healthcare utilisation among children and young people: A retrospective, cross-sectional study of linked healthcare data. BMJ Paediatr Open. 2018;2(1):e000266.

42. Vostanis P, Svirydzenka N, Dugard P, Singh S, Dogra N. Mental health service use by adolescents of Indian and White origin. Arch Dis Child. 2013 Oct 1;98(10):764 LP – 767.

43. Markkula N, Cabieses B, Lehti V, Uphoff E, Astorga S, Stutzin F. Use of health services among international migrant children - a systematic review. Global Health. 2018;14(1).

44. Alkahtani S, Cherrill J, Millward C, Grayson K, Hilliam R, Sammons H, et al. Access to medicines by child refugees in the east midlands region of england: A cross-sectional study. BMJ Open. 2014;4(12).

45. Gkiouleka A, Wong G, Sowden S, Bambra C, Siersbaek R, Manji S, et al. Reducing health inequalities through general practice. Lancet Public Heal. 2023 Jun 1;8(6):e463–72.

46. Sripa P, Hayhoe B, Garg P, Majeed A, Greenfield G. Impact of GP gatekeeping on quality of care, and health outcomes, use, and expenditure: a systematic review. Br J Gen Pract.

For peer review only

2019;69(682):E294–303.

47. Greater Manchester Poverty Action. Greater Manchester Poverty Monitor 2022 [Internet]. 2022 [cited 2023 Apr 5]. Available from: https://www.gmpovertyaction.org/poverty-monitor-2022/

48. The King's Fund. The NHS's role in tackling poverty: Awareness, action and advocacy. 2021.

49. The King's Fund. Poverty and the health and care system: The role of data and partnership in bringing change. 2022.

50. The King's Fund. Understanding pressures in general practice. 2016.

Figures

Figure 1. PRISMA Flow Diagram: associations between CYP or caregiver characteristics and primary care use.

Figure 2. Frequency distribution of CYP ages included in eligible studies.

For peer review only

For peer review only - http://bmjopen.bmj.com/site/about/guidelines.xhtml

Table 1. Outcomes reported in included studies.

	Health services										Dental			
	Attendances/Consultations					Other								
Author (year)	Primary care (GP/other)	Outpatients	Emergency Department/ A&E	Hospital (Elective)	Specialist referrals	Medication (Prescription/OTC)	GP registration	Treatment escalation	Immunisation	Non-medical services*	Dental attendances	Dental registration	Dental/Oral health	Reimbursement
Coughlan *et al*. (2022)	■	■	■	■										
Beaney *et al*. (2021)	■	■	■	■										
de Lusignan *et al*. (2021)	■				■ †			■						
Hasan & West (2021)	■					■								
Hope *et al*. (2021)	■	■	■	■	■ ‡	■								
Manikam *et al*. (2020)	■		■	■		■								
Perry *et al*. (2020)									■					
Ruzangi *et al*. (2020)	■	■	■	■										
Edbrooke-Childs & Patalay (2019)					■ §									
Salomon-Ibarra *et al*. (2019)											■			
Bishop *et al*. (2018)	■	■	■	■	■									
Dreyer *et al*. (2018)	■	■	■	■										
West *et al*. (2018)	■	■	■	■		■								
Cecil *et al*. (2016)			■	■										
Edbrooke-Childs *et al*. (2016)					■ §									
Layte & Nolan (2015)	■													
Alkahtani *et al*. (2014)	■					■	■							
Vostanis *et al*. (2013)	■									■				
Marcenes *et al*. (2013)													■	
Potter *et al*. (2012)										■				
Telford & O'Neill (2012)												■		
Telford *et al*. (2012)												■		■

Abbreviations: GP, general practice.

* Schools and special educational needs services, social services, or youth justice services.

† Dermatologist, GP with a specialty interest in dermatology, or dermatology specialist nurse.

‡ Not specified.

§ Mental health services referrals.

For peer review only

Data availability statement

All data relevant to the study are included in the article or uploaded as supplementary information

Funding statement

This study is funded by the National Institute for Health and Social Care Research (NIHR) through the Children and Families Policy Research Unit (PR-PRU-1217-21301). The views expressed are those of the authors and not necessarily those of the NIHR or the Department of Health and Social Care.

Ethics approval

As a scoping review, this research did not involve participants and thus ethics approval was not required.

Competing interests statement

The authors of this paper declare no conflicts of interest.

Contributor statement

We attest that all authors contributed significantly to the creation of this manuscript, each having fulfilled criteria as established by the ICMJE. Specifically:

Kevin Herbert: Substantial contribution to the design of the work, the acquisition, analysis and interpretation of data for the paper, and the drafting and revision of the submitted paper. They agree to be accountable for all aspects of the work, and in ensuring that questions related to the accuracy or integrity of any part of the work are appropriately investigated and resolved.

Lauren Herlitz: Substantial contribution to the design of the research, the analysis and interpretation of data for the submitted paper, and the critical review and revision of the paper for important intellectual content.

For peer review only - http://bmjopen.bmj.com/site/about/guidelines.xhtml

Jenny Woodman: Substantial contribution to conception and design of the research, the critical review and revision of the paper for important intellectual content, and final approval of the version to be published. They agree to be accountable for all aspects of the work, and in ensuring that questions related to the accuracy or integrity of any part of the work are appropriately investigated and resolved.

Claire Powell: Substantial contribution to the design of the research, the analysis and interpretation of data for the submitted paper, and the critical review and revision of the paper for important intellectual content.

Stephen Morris: Substantial contribution to conception and design of the research, the analysis and interpretation of data for the submitted paper, the critical review and revision of the work for important intellectual content, and final approval of the version to be published. They agree to be accountable for all aspects of the work, and in ensuring that questions related to the accuracy or integrity of any part of the work are appropriately investigated and resolved.

We as authors also confirm that the manuscript has been read and approved by all named authors, and that the order of authors listed in the manuscript has been approved by all named authors.

For peer review only - http://bmjopen.bmj.com/site/about/guidelines.xhtml

For peer review only

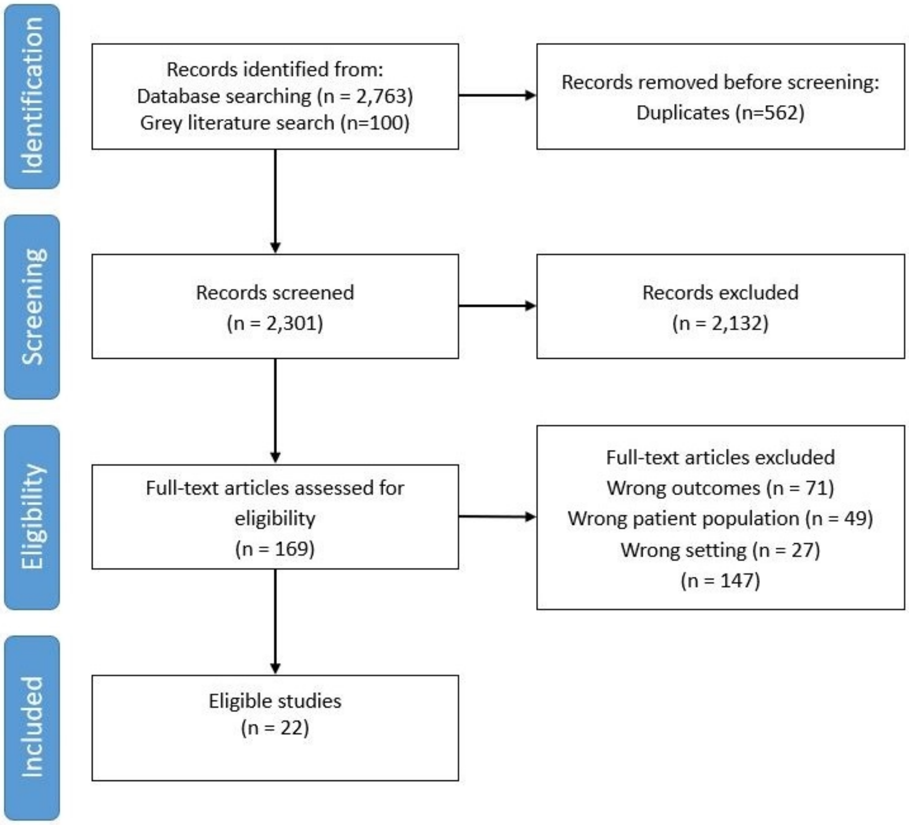

Figure 1. PRISMA Flow Diagram: associations between CYP or caregiver characteristics and primary care use.

208x179mm (96 x 96 DPI)

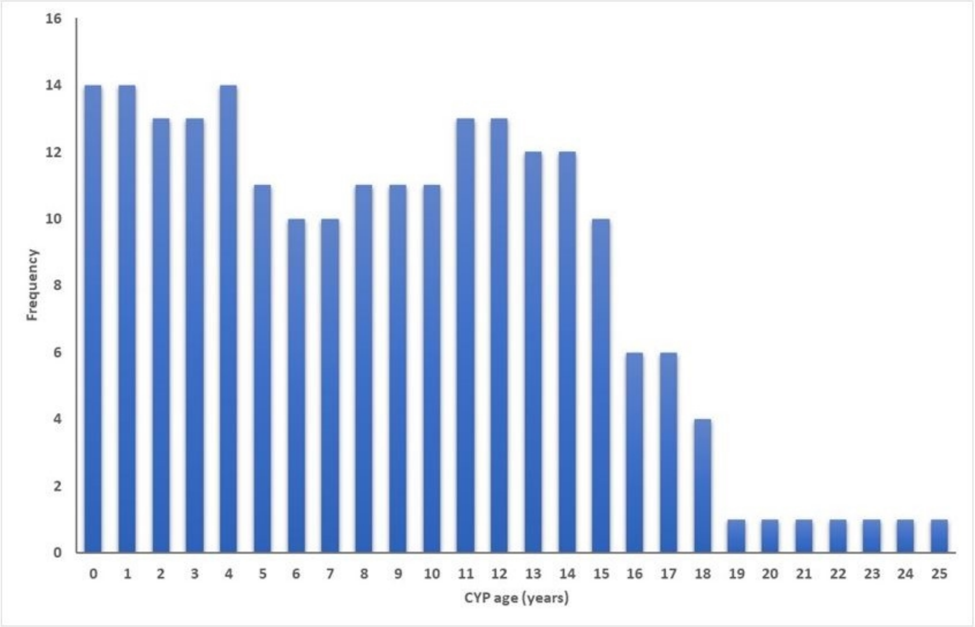

Figure 2. Frequency distribution of CYP ages included in eligible studies.

229x148mm (96 x 96 DPI)

Patient and caregiver characteristics associated with differential use of primary care for children and young people in the UK: a scoping review

Appendices

Appendix A: Search terms and results

Ovid

- Embase 1996 to 2022 Week 24
- Ovid MEDLINE(R) 1996 to June Week 2 2022

Search conducted 23rd June 2022

	Search	Terms	Hits
**1**	Service access	exp "Health Services Accessibility"/ or "access to health services".tw. or "access to care".tw. or exp "Health Equity"/ or "health equity".tw. or exp "Health Literacy"/ or "health literacy".tw. or "healthcare use".tw. or "healthcare utilisation".tw. or "healthcare utilization".tw. or inequality.tw. or inequalities.tw. or equality.tw. or disparity.tw. or disparities.tw. or unequal.tw. or gap*.tw. or gradients.tw. or disadvantage.tw.	1,076,198
**2**	Primary care	exp "Primary Health Care"/ or "primary health care".tw. or "primary care".tw. or exp "Primary Care Nursing"/ or exp "Physicians, Primary Care"/ or exp "General Practice"/ or "general practice*".tw. or "GP surger*".tw. or exp "General Practice, Dental"/ or dentist*.tw. or exp "General Practitioners"/ or "general practitioner*".tw. or exp "Community Pharmacy Services"/ or "community pharmac*".tw.	766,436
**3**	CYP	exp Child/ or child*.tw. or exp Adolescent/ or adolescen*.tw. or exp Infant/ or infant*.tw. or "young people".tw. or youth.tw. or juvenile*.tw. or teenager*.tw. or exp "Parent-Child Relations"/ or "young adult*".tw. or paediatri*.tw.	6,208,476
**4**	UK	("United Kingdom" or England or Wales or Scotland or "Northern Ireland" or "N. Ireland").af.	13,100,534
**5**	Quantitiative studies	(quantit* or consultation* or episode* or visit* or longitudin* or patient-level).af.	4,071,128
**6**		1 and 2 and 3 and 4 and 5	1,932
**7**	Limit to human studies	limit 6 to humans	1,915
**8**	Limit to years 2010 - 2022	limit 7 to yr="2010 - 2022"	1,427
**9**	Deduplication<br><br>Preferences:	remove duplicates from 8<br><br>Deduplication (Ovid)<br>Has full text; Embase > Ovid MEDLINE(R)	1,097

For peer review only - http://bmjopen.bmj.com/site/about/guidelines.xhtml

Scopus

Search conducted 23rd June 2022

Search		Terms	Hits
1	Service access	INDEXTERMS("Health Services Accessibility") OR TITLE-ABS("access to health services") OR TITLE-ABS("access to care") OR INDEXTERMS("Health Equity") OR TITLE-ABS("health equity") OR INDEXTERMS("Health Literacy") OR TITLE-ABS("health literacy") OR TITLE-ABS("healthcare use") OR TITLE-ABS("healthcare utilisation") OR TITLE-ABS("healthcare utilization") OR TITLE-ABS(inequality) OR TITLE-ABS(inequalities) OR TITLE-ABS(equality) OR TITLE-ABS(disparity) OR TITLE-ABS(disparities) OR TITLE-ABS(unequal) OR TITLE-ABS(gap*) OR TITLE-ABS(gradients) OR TITLE-ABS(disadvantage)	2,671,314
2	Primary care	INDEXTERMS("Primary Health Care") OR TITLE-ABS("primary health care") OR TITLE-ABS("primary care") OR INDEXTERMS("Primary Care Nursing") OR INDEXTERMS("Physicians, Primary Care") OR INDEXTERMS("General Practice") OR TITLE-ABS("general practice*") OR TITLE-ABS("GP surger*") OR INDEXTERMS("General Practice, Dental") OR TITLE-ABS(dentist*) OR INDEXTERMS("General Practitioners") OR TITLE-ABS("general practitioner*") OR INDEXTERMS("Community Pharmacy Services") OR TITLE-ABS("community pharmac*")	481,759
3	CYP	INDEXTERMS(Child) OR TITLE-ABS(child*) OR INDEXTERMS(Adolescent) OR TITLE-ABS(adolescen*) OR INDEXTERMS(Infant) OR TITLE-ABS(infant*) OR TITLE-ABS("young people") OR TITLE-ABS(youth) OR TITLE-ABS(juvenile*) OR TITLE-ABS(teenager*) OR INDEXTERMS("Parent-Child Relations") OR TITLE-ABS("young adult*") OR TITLE-ABS(paediatri*)	5,664,633
4	UK	ALL("United Kingdom") OR ALL(England) OR ALL(Wales) OR ALL(Scotland) OR ALL("Northern Ireland") OR ALL("N. Ireland")	12,674,770
5	Quantitiative studies	ALL(quantit* OR consultation* OR episode* OR visit* OR longitudin* OR patient-level)	11,926,797
6	Combine 1-5	#1 AND #2 AND #3 AND #4 AND #5	2,018
7	Limit to articles or reviews	LIMIT-TO (DOCTYPE, "ar") OR LIMIT-TO (DOCTYPE, "re")	1,958
8	Limit to years 2010 - 2022	LIMIT-TO (PUBYEAR, 2022) OR LIMIT-TO (PUBYEAR, 2021) OR LIMIT-TO (PUBYEAR, 2020) OR LIMIT-TO (PUBYEAR, 2019) OR LIMIT-TO (PUBYEAR, 2018) OR LIMIT-TO (PUBYEAR, 2017) OR LIMIT-TO (PUBYEAR, 2016) OR LIMIT-TO (PUBYEAR, 2015) OR LIMIT-TO (PUBYEAR, 2014) OR LIMIT-TO (PUBYEAR, 2013) OR LIMIT-TO (PUBYEAR, 2012) OR LIMIT-TO (PUBYEAR, 2011) OR LIMIT-TO (PUBYEAR, 2010)	1,372
9	Limit to English language publications	LIMIT-TO (LANGUAGE,"English")	1,354

For peer review only - http://bmjopen.bmj.com/site/about/guidelines.xhtml

Web of Science

Web of Science Core Collection: All editions

Search conducted 22nd June 2022

	Search	Terms	Hits
**1**	Service access	ALL=("Health Services Accessibility" OR "access to health services" OR "access to care" OR "Health Equity" OR "health equity" OR "Health Literacy" OR "health literacy" OR "healthcare use" OR "healthcare utilisation" OR "healthcare utilization" OR inequality OR inequalities OR equality OR disparity OR disparities OR unequal OR gap* OR gradients OR disadvantage)	2,284,588
**2**	Primary care	ALL=("Primary Health Care" OR "primary health care" OR "primary care" OR "Primary Care Nursing" OR "Physicians, Primary Care" OR "General Practice" OR "general practice*" OR "GP surger*" OR "General Practice, Dental" OR dentist* OR "General Practitioners" OR "general practitioner*" OR "Community Pharmacy Services" OR "community pharmac*")	444,274
**3**	CYP	ALL=(Child OR child* OR Adolescent OR adolescen* OR Infant OR infant* OR "young people" OR youth OR juvenile* OR teenager* OR "Parent-Child Relations" OR "young adult*" OR paediatri*)	4,378,563
**4**	UK	ALL=("United Kingdom" OR England OR Wales OR Scotland OR "Northern Ireland" OR "N. Ireland")	6,796,242
**5**	Quantitiative studies	ALL=(quantit* OR consultation* OR episode* OR visit* OR longitudin* OR patient-level)	3,328,555
**6**		#1 AND #2 AND #3 AND #4 AND #5	408
**7**	Limit to years 2010 - 2022	#6<br>Publication date: 2010-01-01 to 2022-01-01	315
**8**	Limit to articles or reviews	#7 AND DT=(Article OR Review)	313
**9**	Limit to English language publications	(#8) AND LA=(English)	312

Google

Grey literature search

Search conducted 5th July 2022

Search terms

(site:.org.uk OR site:.gov.uk) AND General Practice* OR General Practitioner* OR Primary Care OR GP* AND Inequality OR Inequalities

For peer review only

Appendix B: Characteristics of included studies (Healthcare need indicator variables for the characteristics of CYP and their caregivers in bold).

**Author (year)** ***sample location***	**Design**	**Health care setting; primary focus of study**	**Sample population**	**Age of sample**	**CYP/caregiver characteristics examined**		**MMAT Score**
					**Sample subset(s)**	**Controlling variable(s)**	
Coughlan *et al*. (2022) *England*	Retrospective cohort study	*General practice, outpatients, emergency department, hospital admissions*: Differences in health care use by health care setting, level of deprivation and ethnic group.	1,484,455 (from 408 GP practices)	0 – 14 years	Ethnicity, deprivation (IMD)	-	****
Beaney *et al*. (2021) *England*	Retrospective cohort study	*General practice, outpatients, emergency department, hospital admissions*: Patterns of healthcare utilisation in the CYP population in Northwest London	378,309	0 – 15 years	**Age**, sex, ethnicity, deprivation (IMD)	-	***** §
de Lusignan *et al*. (2021) *England*	Population-based cohort study	*General practice, specialist referrals:* Use of services and treatment for eczema patients	53,780	0 – 18 years	**Age**, sex, ethnicity, deprivation (IMD), rural-urban classification	-	***** §
Hasan & West (2021) *England*	Prospective cohort study	*Primary care consultations (not specified):* Effects of obesity and ethnicity	7,815 (3,469 White British, 4,346 Pakistani)	8 years	Ethnicity	**Age**, sex, ethnicity, deprivation (not specified), **birthweight**, **gestational age**, maternal BMI, maternal age	*****
Hope *et al*. (2021) *England*	Retrospective cohort study	*General practice, outpatients, emergency department, hospital admissions*: Children exposed to maternal mental illness	489,255	0 – 17 years	**Age**, Ethnicity	Deprivation (IMD)	***** §
Manikam *et al*. (2020) *England*	Retrospective cohort study	*General practice, hospital admissions*: Respiratory tract infection-related healthcare utilisation in children with Down's syndrome	5,866 (992, Down's syndrome, 4,874 control)	0 – 18 years	**Age**, sex, **comorbidities**, **respiratory tract infection type**	-	***** §
Perry *et al*. (2020) *Wales*	Routine health data linked to birth cohort	*General practice (vaccinations)*: Association of deprivation with pertussis vaccination uptake	163,733	0 – 1 years	Deprivation (Welsh IMD)	-	****
Ruzangi *et al*. (2020) *England*	Population-based cohort study	*General practice, outpatients, emergency department, hospital admissions*: Analysis of individual-level patient data to derive population-based estimates of healthcare utilisation	1,484,455 (from 408 GP practices)	0 – 14 years	**Age**	-	***** §
Edbrooke-Childs & Patalay (2019) *England*	Routine youth mental health services data	*General practice, emergency department, mental health services, child health services, education services*: Referral routes to youth mental health services	14,588	0 – 25 years (0-5, 6-12, 13-25 years)	-	**Age**, sex, ethnicity, deprivation (IDACI), **problem type**, contextual factors (home life, school, community, engagement)	*****
Salomon-Ibarra *et al*. (2019) *England*	Routine health data linked to birth cohort	*NHS England dental services:* Dental attendance	155,308	0 – 1 year	-	Deprivation (IMD)	****

For peer review only - http://bmjopen.bmj.com/site/about/guidelines.xhtml

Bishop *et al*. (2018) *England*	Prospective birth cohort study	*General practice, outpatients, emergency department, hospital admissions*: Children with congenital abnormalities on utilisation of primary and secondary care and referrals to specialists.	13,857	0 – 4 years	-	Deprivation (Means-tested benefit status), ethnicity, maternal age, maternal educational attainment, consanguinity, **multimorbidity**	*****
Dreyer *et al*. (2018) *England*	Retrospective, cross-sectional study	*General practice, outpatients, emergency department, hospital admissions*: Parental mental health and other family factors	25,252	0 – 15 years	-	**Age**, sex, deprivation (IMD), **CYP long-term conditions** Parent: age, healthcare utilisation, long-term conditions Household: Number of adults aged 18–55 years, number of CYP aged 0–15 years, presence of any patient aged ≥65 years, total number of patients in household	*****
West *et al*. (2018) *England*	Prospective cohort study	*General practice, outpatients, emergency department, hospital admissions*: Early childhood morbidity and small size at birth	8,850	0 – 3 years	Ethnicity	Sex, maternal parity, **gestational age**, maternal age, social economic information (maternal education, housing tenure, means-tested bene- fits), smoking	*****
Cecil *et al*. (2016) *England*	Cross-sectional, population-based study	*General practice, emergency department, hospital admissions*: Primary care access, emergency department visits, and unplanned short hospitalizations	9.5 million	0 – 15 years	-	Practice deprivation (IMD), urban/rural profile, percentage of the registered population aged ≥65 years	****
Edbrooke-Childs *et al*. (2016) *England*	Routinely collected child and adolescent mental health services data	*General practice, emergency department, social services, education services, child health services, youth justice, self-referral, other services:* Children with mental health problems	11,592 (26 CAMHS)	0 – 18 years	-	**Age**, sex, ethnicity	*****
Layte & Nolan (2015) *Ireland and Scotland*	Child cohort studies	*General practice:* Use of GP services	9,719	2 and 4 years	-	**Age**, sex, deprivation (family income) Healthcare need: **Birthweight**, **gestation**, **parental assessment of child's general health status**, **exposure to accidents** Non-need: Number of siblings, maternal highest level of education, maternal employment status, household composition, maternal ethnicity Health care entitlements	*****

For peer review only

For peer review only - http://bmjopen.bmj.com/site/about/guidelines.xhtml

Alkahtani *et al*. (2014) *England*	Cross-sectional study	*General practice, over the counter medication:* Access to medicines by child refugees	216 (117 refugee, 99 control)	4 – 5 years	-	-	***
Vostanis *et al*. (2013) *England* †	Cross-sectional study	*General practice, CAMHS and paediatric hospital services*: Difference in level of need and service utilisation by young ethnic minority groups.	2,900	13 – 15 years	-	**Age**, ethnicity, free school meals, abnormal score (SDQ or MFQ-short), who the child lives with	*****
Marcenes *et al*. (2013) *England*	Cluster sampled, cross-sectional study	*Dental health (British Association for the Study of Community Dentistry):* Oral health	1,285	3 – 4 years	Deprivation (IMD)	Sex, ethnicity, London borough	****
Potter *et al*. (2012) *UK*	Longitudinal cohort study	General practice, paediatrics, mental health services, social services, youth justice: Mental disorder in children of parents with depression	333	9 – 17 years	**Age**, sex, parental age, family status, parental problem drinking, **child comorbidity (≥2 disorders)**, **child suicidality/ self-harm**, lack of parental concern, parental DSM-IV current depressive episode, frequent parental depressive episodes (≥4)	-	**** §
Telford & O'Neill (2012) *Northern Ireland*	Longitudinal cohort study	*General Dental Service (Northern Ireland):* Dental health investment	13,564	11 – 12 years	**Age**	Sex, deprivation (NS-SEC), highest educational attainment, parental marital status, number of siblings	***** §
Telford *et al*. (2012) *Northern Ireland*	Longitudinal cohort study	*General Dental Service (Northern Ireland):* Provision of NHS general dental practitioner care	12,846	11 – 12 years	-	Highest educational attainment, parental marital status, number of siblings, orthodontic treatment	****

† Identified from Markkula *et al*., 2018.

‡ MMAT score: ***** high quality; **** high quality; *** average quality; ** low quality; * low quality; No stars, poor quality.

§ Indicator of healthcare need used to define study sample only.

Abbreviations: CAMHS, child and adolescent mental health services; CYP, children and young people; GP, general practice; IDACI, Income Deprivation Affecting Children Index; IMD, Index of Multiple Deprivation; MFQ-short, Mood and Feelings Questionnaire-short; MMAT, Mixed Methods Appraisal Tool; NHS, National Health Service; NS-SEC, National statistics socio-economic classification of occupation; SDQ, Strengths and Difficulties Questionnaire.

For peer review only - http://bmjopen.bmj.com/site/about/guidelines.xhtml

Appendix C: Mixed Methods Appraisal Tool (MMAT) quality assessment of eligible studies

Criteria scoring: 0 = Not met; 1 = Partially met; 2 = Fully met.

Total score (Max 14): * ≤5; ** 6-9; *** 10-11; **** 12-13; ***** 14.

		Publication										
Category of study designs	Methodological quality criteria	Coughlan *et al.*, 2022	Beaney *et al.*, 2021	de Lusignan *et al.*, 2021	Hasan & West, 2021	Hope *et al.*, 2021	Manikam *et al.*, 2020	Perry *et al.*, 2020	Ruzangi *et al.*, 2020	Edbrooke-Childs & Patalay, 2019	Salomon-Ibarra *et al.*, 2019	Bishop *et al.*, 2018
Screening questions (for all types)	S1. Are there clear research questions?	2	2	2	2	2	2	2	2	2	2	2
	S2. Do the collected data allow to address the research questions?	2	2	2	2	2	2	2	2	2	2	2
Further appraisal may not be feasible or appropriate when the answer is 'No' or 'Can't tell' to one or both screening questions.												
Quantitative non-randomized controlled trials	3.1. Are the participants representative of the target population?					2						2
	3.2. Are measurements appropriate regarding both the outcome and intervention (or exposure)?					2						2
	3.3. Are there complete outcome data?					2						2
	3.4. Are the confounders accounted for in the design and analysis?					2						2
	3.5. During the study period, is the intervention administered (or exposure occurred) as intended?					2						2
Quantitative descriptive	4.1. Is the sampling strategy relevant to address the research question?	2	2	2	2		2	2	2	2	2	
	4.2. Is the sample representative of the target population?	2	2	2	2		2	2	2	2	2	
	4.3. Are the measurements appropriate?	2	2	2	2		2	2	2	2	2	
	4.4. Is the risk of nonresponse bias low?	2	2	2	2		2	2	2	2	2	
	4.5. Is the statistical analysis appropriate to answer the research question?	2	2	2	2		2	2	2	2	2	
Total score		14	14	14	14	14	14	14	14	14	14	14

For peer review only - http://bmjopen.bmj.com/site/about/guidelines.xhtml

		Publication										
Category of study designs	Methodological quality criteria	Dreyer *et al.*, 2018	Vostanis et al., 2013 †	West *et al.*, 2018	Cecil *et al.*, 2016	Edbrooke-Childs *et al.*, 2016	Layte & Nolan, 2015	Alkahtani *et al.*, 2014	Marcenes *et al.*, 2013	Potter *et al.*, 2012	Telford & O'Neill, 2012	(C. Telford *et al.*, 2012)
Screening questions (for all types)	S1. Are there clear research questions?	2	2	2	2	2	2	2	2	2	2	2
	S2. Do the collected data allow to address the research questions?	2	2	2	2	2	2	2	2	2	2	2
Further appraisal may not be feasible or appropriate when the answer is 'No' or 'Can't tell' to one or both screening questions.												
Quantitative non-randomized controlled trials	3.1. Are the participants representative of the target population?				2		2	1				
	3.2. Are measurements appropriate regarding both the outcome and intervention (or exposure)?				2		2	2				
	3.3. Are there complete outcome data?				2		2	2				
	3.4. Are the confounders accounted for in the design and analysis?				2		2	0				
	3.5. During the study period, is the intervention administered (or exposure occurred) as intended?				2		2	2				
Quantitative descriptive	4.1. Is the sampling strategy relevant to address the research question?	2	2	2		2			2	2	2	2
	4.2. Is the sample representative of the target population?	2	2	2		2			2	1	2	2
	4.3. Are the measurements appropriate?	2	2	2		2			2	2	2	2
	4.4. Is the risk of nonresponse bias low?	2	2	2		2			2	2	2	2
	4.5. Is the statistical analysis appropriate to answer the research question?	2	2	2		2			2	2	2	2
Total score		14	14	14	14	14	14	11	14	13	14	14

† Identified from Markkula *et al.*, 2018.

For peer review only - http://bmjopen.bmj.com/site/about/guidelines.xhtml

References

Alkahtani, S., Cherrill, J., Millward, C., Grayson, K., Hilliam, R., Sammons, H., & Choonara, I. (2014). Access to medicines by child refugees in the east midlands region of england: A cross-sectional study. *BMJ Open*, *4*(12). https://doi.org/10.1136/bmjopen-2014-006421

Beaney, T., Clarke, J., Woodcock, T., McCarthy, R., Saravanakumar, K., Barahona, M., Blair, M., & Hargreaves, D. S. (2021). Patterns of healthcare utilisation in children and young people: A retrospective cohort study using routinely collected healthcare data in Northwest London. *BMJ Open*, *11*(12). https://doi.org/10.1136/bmjopen-2021-050847

Bishop, C., Small, N., & Parslow, R. (2018). Healthcare use for children with complex needs: Using routine health data linked to a multiethnic, ongoing birth cohort. *BMJ Open*, *8*(3), e018419. https://doi.org/http://dx.doi.org/10.1136/bmjopen-2017-018419

Cecil, E., Bottle, A., Cowling, T. E., Majeed, A., Wolfe, I., & Saxena, S. (2016). Primary care access, emergency department visits, and unplanned short hospitalizations in the UK. *Pediatrics*, *137*(2). https://doi.org/10.1542/peds.2015-1492

Coughlan, C., Ruzangi, J., Neale, F., Nezafat Maldonado, B., Blair, M., Bottle, A., Saxena, S., & Hargreaves, D. (2022). Social and ethnic group differences in healthcare use by children aged 0-14 years: A population-based cohort study in England from 2007 to 2017. *Archives of Disease in Childhood*, *107*(1), 32–39. https://doi.org/https://dx.doi.org/10.1136/archdischild-2020-321045

de Lusignan, S., Alexander, H., Broderick, C., Dennis, J., McGovern, A., Feeney, C., & Flohr, C. (2021). Patterns and trends in eczema management in UK primary care (2009-2018): A population-based cohort study. *Clinical and Experimental Allergy*, *51*(3), 483–494. https://doi.org/http://dx.doi.org/10.1111/cea.13783

Dreyer, K., Williamson, R., Hargreaves, D., & Rosen, R. (2018). Associations between parental mental health and other family factors and healthcare utilisation among children and young people: A retrospective, cross-sectional study of linked healthcare data. *BMJ Paediatrics Open*, *2*(1), e000266. https://doi.org/https://dx.doi.org/10.1136/bmjpo-2018-000266

Edbrooke-Childs, J., Newman, R., Fleming, I., Deighton, J., & Wolpert, M. (2016). The association between ethnicity and care pathway for children with emotional problems in routinely collected child and adolescent mental health services data. *European Child and Adolescent Psychiatry*, *25*(5), 539–546. https://doi.org/10.1007/s00787-015-0767-4

Edbrooke-Childs, J., & Patalay, P. (2019). Ethnic Differences in Referral Routes to Youth Mental Health Services. *Journal of the American Academy of Child and Adolescent Psychiatry*, *58*(3), 368-375.e1. https://doi.org/https://dx.doi.org/10.1016/j.jaac.2018.07.906

Hasan, T., & West, J. (2021). Body mass index and use and costs of primary care services among white british and pakistani children: Findings from the born in bradford cohort study. *Archives of Disease in Childhood*, *106*(SUPPL 1), A393–A394. https://doi.org/http://dx.doi.org/10.1136/archdischild-2021-rcpch.683

Hope, H., Osam, C., Kontopantelis, E., Hughes, S., Munford, L., Ashcroft, D., & Pierce, M. (2021). The healthcare resource impact of maternal mental illness on children and adolescents: UK retrospective cohort study. *British Journal of Psychiatry*, *219*(3), 515–522. https://doi.org/https://dx.doi.org/10.1192/bjp.2021.65

Layte, R., & Nolan, A. (2015). Income-related inequity in the use of GP services by children: a comparison of Ireland and Scotland. *The European Journal of Health Economics : HEPAC : Health Economics in Prevention and Care*, *16*(5), 489–506.

For peer review only

https://doi.org/https://dx.doi.org/10.1007/s10198-014-0587-3

Manikam, L., Schilder, A., Lakhanpaul, M., Littlejohns, P., & Alexander, E. (2020). Respiratory tract infection-related healthcare utilisation in children with Down's syndrome. *Infection*, *48*(3), 403–410. https://doi.org/http://dx.doi.org/10.1007/s15010-020-01408-5

Marcenes, W., Muirhead, V. E., Murray, S., Redshaw, P., Bennett, U., & Wright, D. (2013). Ethnic disparities in the oral health of three- to four-year-old children in East London. *British Dental Journal*, *215*(2). https://doi.org/10.1038/sj.bdj.2013.687

Markkula, N., Cabieses, B., Lehti, V., Uphoff, E., Astorga, S., & Stutzin, F. (2018). Use of health services among international migrant children - a systematic review. *Globalization and Health*, *14*(1). https://doi.org/10.1186/s12992-018-0370-9

Perry, M., McGowan, A., Roberts, R., & Cottrell, S. (2020). Timeliness and equity of infant pertussis vaccination in wales: Analysis of the three dose primary course. *Vaccine*, *38*(6), 1402–1407. https://doi.org/https://dx.doi.org/10.1016/j.vaccine.2019.12.001

Potter, R., Mars, B., Eyre, O., Legge, S., Ford, T., Sellers, R., Craddock, N., Rice, F., Collishaw, S., Thapar, A., & Thapar, A. K. (2012). Missed opportunities: Mental disorder in children of parents with depression. *British Journal of General Practice*, *62*(600), e487–e493. https://doi.org/10.3399/bjgp12X652355

Ruzangi, J., Blair, M., Cecil, E., Greenfield, G., Bottle, A., & Hargreaves, D. (2020). Trends in healthcare use in children aged less than 15 years: A population-based cohort study in England from 2007 to 2017. *BMJ Open*, *10*(5), e033761. https://doi.org/http://dx.doi.org/10.1136/bmjopen-2019-033761

Salomon-Ibarra, C., Ravaghi, V., Hill, K., Jones, C., Landes, D., & Morris, A. (2019). Low rates of dental attendance by the age of one and inequality between local government administrative areas in England. *Community Dental Health*, *36*(1), 22–26. https://doi.org/10.1922/CDH_4390Salomon-Ibarra05

Telford, C. J., & O'Neill, C. (2012). Summary of: Changes in dental health investment across the adolescent years. *British Dental Journal*, *212*(9), 438–439. https://doi.org/10.1038/sj.bdj.2012.372

Telford, C., Murray, L., Donaldson, M., & O'Neill, C. (2012). An analysis examining socio-economic variations in the provision of NHS general dental practitioner care under a fee for service contract among adolescents: Northern Ireland Longitudinal Study. *Community Dentistry and Oral Epidemiology*, *40*(1), 70–79. https://doi.org/http://dx.doi.org/10.1111/j.1600-0528.2011.00649.x

Vostanis, P., Svirydzenka, N., Dugard, P., Singh, S., & Dogra, N. (2013). Mental health service use by adolescents of Indian and White origin. *Archives of Disease in Childhood*, *98*(10), 764 LP – 767. https://doi.org/10.1136/archdischild-2013-303772

West, J., Kelly, B., Collings, P., Santorelli, G., & Mason, D. (2018). Is small size at birth associated with early childhood morbidity in white British and Pakistani origin UK children aged 0-3? Findings from the born in Bradford cohort study. *BMC Pediatrics*, *18*(1), 22. https://doi.org/http://dx.doi.org/10.1186/s12887-018-0987-0

For peer review only

Preferred Reporting Items for Systematic reviews and Meta-Analyses extension for Scoping Reviews (PRISMA-ScR) Checklist

SECTION	ITEM	PRISMA-ScR CHECKLIST ITEM	REPORTED ON PAGE #
**TITLE**			
Title	1	Identify the report as a scoping review.	1
**ABSTRACT**			
Structured summary	2	Provide a structured summary that includes (as applicable): background, objectives, eligibility criteria, sources of evidence, charting methods, results, and conclusions that relate to the review questions and objectives.	3
**INTRODUCTION**			
Rationale	3	Describe the rationale for the review in the context of what is already known. Explain why the review questions/objectives lend themselves to a scoping review approach.	5
Objectives	4	Provide an explicit statement of the questions and objectives being addressed with reference to their key elements (e.g., population or participants, concepts, and context) or other relevant key elements used to conceptualize the review questions and/or objectives.	6
**METHODS**			
Protocol and registration	5	Indicate whether a review protocol exists; state if and where it can be accessed (e.g., a Web address); and if available, provide registration information, including the registration number.	6
Eligibility criteria	6	Specify characteristics of the sources of evidence used as eligibility criteria (e.g., years considered, language, and publication status), and provide a rationale.	6
Information sources*	7	Describe all information sources in the search (e.g., databases with dates of coverage and contact with authors to identify additional sources), as well as the date the most recent search was executed.	7
Search	8	Present the full electronic search strategy for at least 1 database, including any limits used, such that it could be repeated.	7
Selection of sources of evidence†	9	State the process for selecting sources of evidence (i.e., screening and eligibility) included in the scoping review.	7
Data charting process‡	10	Describe the methods of charting data from the included sources of evidence (e.g., calibrated forms or forms that have been tested by the team before their use, and whether data charting was done independently or in duplicate) and any processes for obtaining and confirming data from investigators.	7
Data items	11	List and define all variables for which data were sought and any assumptions and simplifications made.	7
Critical appraisal of individual sources of evidence§	12	If done, provide a rationale for conducting a critical appraisal of included sources of evidence; describe the methods used and how this information was used in any data synthesis (if appropriate).	7

St. Michael's
Inspired Care. Inspiring Science.

For peer review only - http://bmjopen.bmj.com/site/about/guidelines.xhtml

SECTION	ITEM	PRISMA-ScR CHECKLIST ITEM	REPORTED ON PAGE #
Synthesis of results	13	Describe the methods of handling and summarizing the data that were charted.	8
**RESULTS**			
Selection of sources of evidence	14	Give numbers of sources of evidence screened, assessed for eligibility, and included in the review, with reasons for exclusions at each stage, ideally using a flow diagram.	8
Characteristics of sources of evidence	15	For each source of evidence, present characteristics for which data were charted and provide the citations.	8
Critical appraisal within sources of evidence	16	If done, present data on critical appraisal of included sources of evidence (see item 12).	9
Results of individual sources of evidence	17	For each included source of evidence, present the relevant data that were charted that relate to the review questions and objectives.	10
Synthesis of results	18	Summarize and/or present the charting results as they relate to the review questions and objectives.	N/A (See p8)
**DISCUSSION**			
Summary of evidence	19	Summarize the main results (including an overview of concepts, themes, and types of evidence available), link to the review questions and objectives, and consider the relevance to key groups.	15
Limitations	20	Discuss the limitations of the scoping review process.	16
Conclusions	21	Provide a general interpretation of the results with respect to the review questions and objectives, as well as potential implications and/or next steps.	17
**FUNDING**			
Funding	22	Describe sources of funding for the included sources of evidence, as well as sources of funding for the scoping review. Describe the role of the funders of the scoping review.	26

JBI = Joanna Briggs Institute; PRISMA-ScR = Preferred Reporting Items for Systematic reviews and Meta-Analyses extension for Scoping Reviews.
* Where *sources of evidence* (see second footnote) are compiled from, such as bibliographic databases, social media platforms, and Web sites.
† A more inclusive/heterogeneous term used to account for the different types of evidence or data sources (e.g., quantitative and/or qualitative research, expert opinion, and policy documents) that may be eligible in a scoping review as opposed to only studies. This is not to be confused with *information sources* (see first footnote).
‡ The frameworks by Arksey and O'Malley (6) and Levac and colleagues (7) and the JBI guidance (4, 5) refer to the process of data extraction in a scoping review as data charting.
§ The process of systematically examining research evidence to assess its validity, results, and relevance before using it to inform a decision. This term is used for items 12 and 19 instead of "risk of bias" (which is more applicable to systematic reviews of interventions) to include and acknowledge the various sources of evidence that may be used in a scoping review (e.g., quantitative and/or qualitative research, expert opinion, and policy document).

From: Tricco AC, Lillie E, Zarin W, O'Brien KK, Colquhoun H, Levac D, et al. PRISMA Extension for Scoping Reviews (PRISMAScR): Checklist and Explanation. Ann Intern Med. 2018;169:467–473. doi: 10.7326/M18-0850.

For peer review only - http://bmjopen.bmj.com/site/about/guidelines.xhtml
